# Network Pharmacology Analysis Reveals Multi-Target Hepatoprotective Mechanisms of a Multi-Component Pharmacopuncture Against Ephedra-Associated Liver Injury with Implications for Mitochondrial Quality Control

**DOI:** 10.3390/medicina62050849

**Published:** 2026-04-29

**Authors:** Ji Hye Hwang, Chul Jung

**Affiliations:** 1 Department of Acupuncture & Moxibustion Medicine, College of Korean Medicine, Gachon University, Seongnam 13120, Republic of Korea; 2 Namsangcheon Korean Medicine Clinic, Seoul 06656, Republic of Korea; jcnu2000@hanmail.net

**Keywords:** drug-induced liver injury, *Ephedra sinica*, network pharmacology, pharmacopuncture, mitophagy

## Abstract

*Background and Objectives:* Drug-induced liver injury (DILI) is increasingly associated with the use of herbal medicines. *Ephedra sinica* (ES) occasionally induces hepatocellular injury, yet therapeutic strategies for herb-induced liver injury are limited. This study investigated the potential mechanisms of a multicomponent pharmacopuncture formulation (VP) in ES-associated hepatotoxicity. *Materials and Methods:* Bioactive constituents of VP were collected from pharmacological databases and literature. The physicochemical properties were evaluated using SwissADME. Compound–target interactions were identified using the STITCH database and integrated with DILI–related genes retrieved from GeneCards (relevance score ≥ 5.0). Protein–protein interaction network analysis, Gene Ontology enrichment, and KEGG pathway analyses were performed. *Results:* A total of 22 overlapping targets were identified. A nine-gene module—comprising TNF, IL6, STAT3, CASP3, PINK1, PRKN, NFE2L2, HMOX1, and ABCB11—was associated with key biological processes, including inflammatory signaling, mitochondrial quality control, oxidative stress regulation, and hepatobiliary transport. *Conclusions:* These findings suggest that VP may modulate multiple biological processes relevant to hepatotoxic stress, including inflammatory signaling, mitochondrial quality control, and bile acid transport. These results provide a plausible mechanistic framework for further investigation, pending experimental validation.

## 1. Introduction

Drug-induced liver injury (DILI) is a clinically significant condition and represents one of the leading causes of acute liver dysfunction associated with both conventional pharmaceuticals and herbal medicines [1,2,3,4,5]. Recent global RUCAM-based analyses have reported that herb-induced liver injury (HILI) accounts for approximately 26% of DILI cases, with higher proportions observed in Asian populations where herbal medicine use is prevalent [6,7]. Among herbal medicines, *Ephedra sinica* (ES) is widely used in respiratory and metabolic disorders [8]. However, ephedrine, its main alkaloid component, has been associated with clinically significant hepatotoxicity in susceptible individuals [9]. The selection of ES-associated hepatotoxicity as the focus of this study was based on several considerations. First, ephedrine-induced DILI has been documented in multiple case reports and pharmacovigilance databases, such as LiverTox, with a clinical pattern of hepatocellular injury [9]. Second, recent toxicological studies suggest a mechanistically distinct profile of ephedrine-induced hepatotoxicity, particularly involving mitochondrial oxidative stress and dysregulated PINK1–PRKN-mediated mitophagy [10,11]. This mechanistic specificity distinguishes ES-associated HILI from other DILI agents and provides a biologically coherent rationale for investigating a multi-target formulation capable of modulating mitochondrial quality control alongside inflammatory and detoxification pathways. Finally, this study was motivated by a clinical observation of ES-associated hepatocellular injury, providing a relevant clinical context for this mechanistic investigation.

The primary management strategy for DILI is immediate discontinuation of the suspected causative agent. According to LiverTox database, although biochemical improvement may begin shortly after drug withdrawal, complete normalization of liver enzymes generally requires 2 to 3 months in cases of hepatocellular injury. In some cases, the injury may persist or even progress despite discontinuation of the drug [12,13]. Therefore, therapeutic strategies that can actively promote hepatic recovery and prevent progression of liver injury are needed.

V-pharmacopuncture (VP) is a multi-component injectable formulation derived from traditional Korean medicine. It comprises animal-derived medicinal materials including Moschus, Fel Ursi, and Calculus Bovis, and several botanical herbs such as Scutellariae Radix, Phellodendri Cortex, Pulsatillae Radix, Sophorae Tonkinensis Radix, and Aucklandiae Radix. In traditional Korean medicine, pharmacopuncture formulations derived from or based on VP are used to treat inflammatory and pain-related conditions [14,15]. This formulation contains several bioactive constituents such as muscone [16], ursodeoxycholic acid (UDCA), tauroursodeoxycholic acid (TUDCA) [17,18,19], berberine [20], and baicalin [20], all of which have hepatoprotective properties. Pharmacological studies have demonstrated that these compounds can modulate key cellular pathways involved in liv-er injury, including activation of the NFE2L2 (Nrf2)-mediated antioxidant defense system, suppression of NF-κB-mediated inflammatory signaling, regulation of apoptosis-related pathways, enhancement of mitochondrial quality control, and maintenance of hepatobiliary bile acid transport via ABCB11 [17,18,19,20,21,22]. Ephedrine-induced hepatotoxicity can be due to impaired mitophagy and mitochondrial stress [10,11]. Baicalin has been reported to have hepatoprotective and antioxidative effects in liver injury [21]. However, the multitarget pharmacological mechanisms through which the diverse constituents of VP collectively counteract hepatotoxic processes, particularly those associated with ephedrine exposure, are not completely understood.

Network pharmacology has emerged as a useful systems biology approach for investigating complex interactions between multicomponent herbal formulations and biological targets [23]. By integrating compound–target networks with disease-associated molecular pathways, this approach enables exploration of potential therapeutic mechanisms underlying traditional medical interventions.

Therefore, the present study employed a network pharmacology–based approach to elucidate multi-target hepatoprotective mechanisms of VP in ES-induced liver injury. To provide additional mechanistic context, the molecular profile of VP was interpreted based on representative hepatoprotective herbal formulations which are commonly used in clinical practice. Further, in cases where rapid biochemical recovery was observed within 10 days of VP treatment, network analysis was conducted to understand the clinical significance of the computational predictions. Through this integrated approach, we aimed to provide mechanistic insights into the potential role of VP as a therapeutic agent for HILI.

## 2. Materials and Methods

### 2.1. Identification of Chemical Constituents of VP

The chemical constituents of VP were identified based on its standardized herbal composition. The VP formulation used in this study was manufactured at Namsangcheon Herbal Medicine Dispensary (Yongin, Republic of Korea) under Korean Good Manufacturing Practice (GMP) standards, and consisted of eight medicinal ingredients: Moschus, Fel Ursi, Calculus Bovis, Scutellariae Radix, Phellodendri Cortex, Pulsatillae Radix, Sophorae Tonkinensis Radix, and Aucklandia Radix. The pharmacopuncture formulation used in this study was prepared using a standardized extraction process using aqueous distillation and ethanol extraction of the constituent materials followed by sterile filtration, according to established pharmacopuncture manufacturing procedures. Candidate compounds of each herb were initially collected from the Traditional Chinese Medicine Systems Pharmacology database (TCMSP; https://tcmsp-e.com/tcmsp.php, accessed on 9 February 2026) [24] and PubChem [25]. To ensure structural consistency, duplicate compounds were removed and chemical identifiers were standardized using InChIKey matching. Given the limitations of database-driven retrieval of animal-derived materials, an extensive manual literature search was conducted to identify well-documented bioactive constituents. This curated list ensured the inclusion of experimentally validated compounds such as muscone, UDCA, and TUDCA. After standardization and curation, 158 bioactive compounds (148 plant-derived and 10 manually curated) were identified in the VP compound library. Among these, 144 compounds with retrievable canonical SMILES structures were subjected to physicochemical and ADME profiling using SwissADME [26], and 133 compounds were successfully mapped to the STITCH database (version 5.0) [27] for downstream target prediction.

### 2.2. Physicochemical and ADME Profiling

Physicochemical and pharmacokinetic properties of VP constituents were evaluated using the SwissADME web server [26]. Four key parameters were analyzed to assess drug-likeness and pharmacokinetic feasibility: (1) gastrointestinal (GI) absorption was estimated using the BOILED-Egg model, (2) solubility class was estimated using the Estimated SOLubility (ESOL) model, (3) topological polar surface area (TPSA) with 140 Å^2^ was used as a permeability threshold, and (4) Lipinski’s rule of Five violations. Because VP is administered via pharmacopuncture rather than oral ingestion, these parameters are not intended as pharmacokinetic predictors for the injectable formulation. Rather, they serve as general physicochemical characterization of the constituent library and provide supporting context for the pharmacopuncture delivery rationale: compounds with limited aqueous solubility or predicted low gastrointestinal absorption may benefit from direct injectable administration, which bypasses first-pass metabolism and gastrointestinal absorption barriers. TPSA and solubility data are therefore presented to characterize the physicochemical diversity of the formulation and to contextualize the rationale for pharmacopuncture as a delivery route, rather than to predict oral bioavailability.

### 2.3. Compound–Target Interaction Analysis and Literature Integration

Potential protein targets for VP constituents were retrieved from the STITCH database (version 5.0) [27] using a confidence score threshold of ≥0.400. A higher STITCH confidence threshold (≥0.700) was also explored; however, this resulted in a substantially reduced interaction network with limited coverage for downstream analyses. Therefore, a threshold of ≥0.400 was retained to balance network completeness and specificity. Initially, the curated pool of the identified candidate compounds was screened against the STITCH database using InChIKey and structural identifiers. This process yielded a standardized set of 133 unique compounds that were successfully mapped to the database and used for downstream target prediction. The database-derived targets were supplemented with manual literature curation to ensure the inclusion of biologically validated mechanisms. Publications retrieved from PubMed and Google Scholar were screened to identify experimentally validated molecular targets of the major VP constituents including muscone, TUDCA, and bilirubin. Particular attention was given to the targets involved in hepatoprotective signaling pathways, specifically antioxidant defense and detoxification (e.g., NFE2L2, HMOX1, and ABCB11) [17,21,22], mitochondrial quality control and mitophagy (e.g., PINK1 and PRKN) [9,10,18], apoptosis regulation (e.g., BCL2 and CASP3) [19,21], and inflammatory signaling (e.g., TNF, IL6, and STAT3) [20,22]. This integration of database-derived targets with literature-supported targets allowed a comprehensive representation of the molecular pharmacology of VP, totaling 287 unique protein targets, which were further refined to 71 high-confidence targets for downstream analysis.

### 2.4. Identification of Liver Injury–Related Genes

To obtain a comprehensive list of genes associated with DILI, disease-related targets were retrieved from the GeneCards database (version 5.26) [28]. The following Boolean search terms were used: “drug-induced liver injury” OR “DILI” OR “hepatotoxicity” OR “toxic liver injury” OR “liver toxicity”. Initially, 1464 genes were identified. To ensure sufficient coverage of DILI-relevant targets while excluding very low-confidence associations, genes with a relevance score ≥5.0 were retained, resulting in 109 DILI-associated targets. This threshold was selected to balance specificity and sensitivity, consistent with prior network pharmacology studies of herbal formulations in liver injury. It is acknowledged that this cutoff influences target set composition; a more stringent threshold (≥7.0) yields 64 DILI-associated genes, retaining major targets such as TNF, NFE2L2, HMOX1, and ABCB11, but excluding IL6 (score: 6.8) and STAT3 (score: 5.5). Notably, PINK1 and PRKN are absent from GeneCards DILI results at any threshold, reflecting that these mitophagy regulators were incorporated based on ephedrine-specific experimental evidence [10,11] rather than general DILI database associations—underscoring the complementary role of evidence-based literature curation in this study. Accordingly, the ≥5.0 threshold was retained to preserve biologically meaningful coverage for downstream network analysis, while transparently acknowledging its influence on target composition. By intersecting these disease targets with 287 VP-associated targets identified in Section 2.3, a final set of 22 overlapping therapeutic targets were identified. To bridge these computational findings with the specific clinical context of this study, a focused nine-gene mechanistic module associated with Ephedra-induced hepatotoxicity was curated. This module integrated core hub genes from the overlapping network including inflammatory and apoptosis regulators (TNF, IL6, STAT3, and CASP3) [21,23], toxin-specific regulators for mitochondrial quality control (including PINK1 and PRKN) [9,10], hepatobiliary transport molecule (ABCB11) [22], and antioxidant defense molecules (NFE2L2 and HMOX1) [16,21]. This curated module was used to evaluate whether the VP targets intersected effectively with the molecular pathways involved in Ephedra-associated liver injury.

### 2.5. Protein–Protein Interaction (PPI) Network Construction

The therapeutic targets identified in this study were subjected to PPI analysis using STRING database (version 12.0) [29]. The interaction parameters were set to: (1) species: *Homo sapiens*, and (2) confidence score: ≥0.700 (high confidence). The resulting interaction network was imported into Cytoscape (version 3.10.1) [30] for visualization and topological analysis. The cytoHubba plugin (version 0.1) [31] was used to identify the central regulatory hubs within the network. Nodes were ranked based on the Maximal Clique Centrality (MCC) algorithm, which has been reported to be highly effective for identifying essential proteins in biological networks.

### 2.6. Functional and Pathway Enrichment Analysis

To investigate the biological significance of the identified targets, functional enrichment analysis was performed using the DAVID bioinformatics resource (2021 update) [29]. Two types of enrichment analyses were conducted: (1) Gene ontology (GO) enrichment for biological processes, and (2) Kyoto Encyclopedia of Genes and Genomes (KEGG) pathway analysis. Statistical significance was set at *p*-value < 0.05 and a false discovery rate (FDR) < 0.05. Enriched biological processes and pathways were interpreted in the context of hepatocellular injury mechanisms including inflammation, oxidative stress, mitochondrial dysfunction, apoptosis regulation, and metabolic disturbances.

### 2.7. Clinical Motivation and Comparative Herbal Reference Analysis

This study was motivated by a clinical case of acute hepatocellular injury associated with an Ephedra-containing herbal decoction, in which the patient exhibited markedly elevated liver enzyme levels (ALT: 709 IU/L). Following treatment with VP, the patient’s liver enzyme levels normalized within 10 days. This clinical observation served as the motivating context for the present in silico investigation; however, it cannot be interpreted as validation of the computational findings, as no control condition was available and causality cannot be established from a single case. The study was approved by the Institutional Review Board of Gachon University Gil Korean Medical Hospital, with ethical review waived due to the retrospective nature of the single-patient observation (protocol code GIRB-25-106). To provide mechanistic context for the hepatoprotective effects of VP, four commonly used hepatoprotective herbal prescriptions (HPs) were included as a comparative herbal reference set: Saenggan-geonbi-tang [32], Injinoryeong-san [33], Hwangnyeonhaedok-tang [23], and Yongdamsagan-tang [34] (Table S1). The molecular target profiles of these formulations were analyzed and compared with that of VP to explore the potential overlapping hepatoprotective mechanisms and contextualize multitarget pharmacological actions of VP as a therapeutic drug.

## 3. Results

### 3.1. Identification and Standardization of Bioactive Compounds

#### 3.1.1. Compound Library Construction Through Database Retrieval and Literature Curation

To establish a comprehensive and standardized chemical library for VP, a dual-source compound identification strategy integrating database retrieval and literature review was employed. A total of 148 plant-derived compounds were collected after removing duplicates (e.g., coptisine and quercetin) and standardizing the identifiers. Although TCMSP primarily curates botanical constituents, certain entries related to animal-derived materials (e.g., Bovis Calculus) were observed in the database and these compounds were cross-referenced with established pharmacological literature to ensure their relevance to VP. Recognizing the limitations of database-driven approaches for obtaining pharmacologically critical animal-derived constituents, further extensive literature survey using the PubMed and PMC databases was conducted. Ten additional pharmacological marker compounds were manually curated including representative constituents from Moschus, Fel Ursi, and Bovis Calculus (e.g., muscone, UDCA, TUDCA, cholic acid, bilirubin, taurocholic acid, and glycolic acid) and key bioactive markers such as oleanolic acid, ursolic acid, and ergosterol, which were underrepresented in the initial database search. This resulted in an integrated pool of 158 candidate compounds for standardization (Table S2). All compounds were standardized using InChIKey and SMILES identifiers. After structural standardization and mapping, 133 unique compounds were retained for network pharmacological analysis in the STITCH database. A total of 144 compounds with retrievable canonical SMILES were included in the ADME screening to characterize the physicochemical landscape of the VP formulation (Table 1).

#### 3.1.2. Target Prediction and Evidence-Guided Refinement of the Pharmacological Network

Target identification was performed to capture the multitarget therapeutic potential of VP during hepatotoxic stress. Compound–protein interactions were retrieved from the STITCH database (version 5.0) [24] using medium-confidence threshold (combined score ≥ 0.400). This threshold was selected to balance the specificity and network coverage, allowing inclusion of both direct molecular interactions and functionally associated targets that are commonly used in network pharmacology analyses. Through STITCH-based screening, 57 human protein targets were identified. An evidence-guided refinement step was conducted to enhance disease relevance and compensate for potential gaps in database predictions. Based on experimental evidence linkage to VP, 14 additional targets with experimentally documented roles in hepatoprotection, oxidative stress regulation, apoptosis control, mitochondrial quality control (including PINK1 and PRKN), and bile acid homeostasis were incorporated (Table S6). Representative examples include NFE2L2 and HMOX1 for antioxidant defense, TNF and IL6 for inflammatory signaling, BCL2 and CASP3 for apoptosis regulation, PINK1 and PRKN for mitochondrial quality control, and ABCB11 for hepatobiliary transport. This integrative approach ensured that the final 71-target set included both database-predicted interactions and experimentally supported mechanisms [10,11,16,19,20,21,22,23,35,36] relevant to hepatotoxic stress and recovery. The stepwise prioritization process from the initial compound library to the refined pharmacological targets is summarized in Table 1.

### 3.2. Comparative Network Analysis: Therapeutic Specificity of V-Pharmacopuncture

#### 3.2.1. Landscape of Target Overlap Among VP, Hepatoprotective Formulae, and DILI

**Figure 1:**
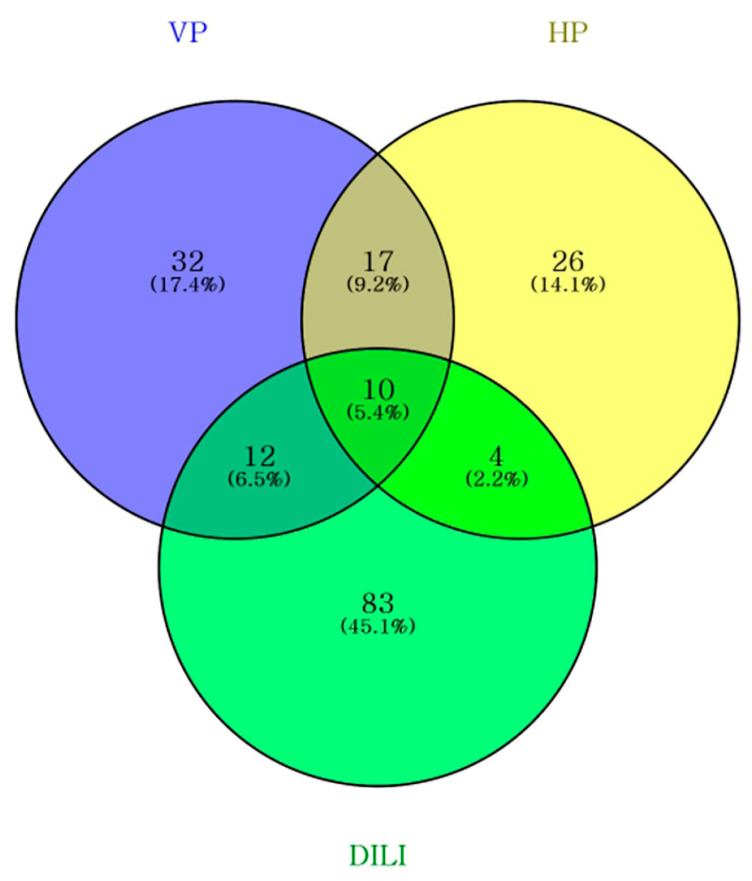
Comparative target overlap among VP, hepatoprotective formulas, and DILI-associated genes. Venn diagram illustrating the distribution of pharmacological targets across VP, conventional hepatoprotective formulae (HP), and drug-induced liver injury (DILI)-associated genes. A total of 71 VP targets, 57 HP targets, and 109 DILI-associated genes (filtered by a relevance score ≥ 5) were compared using identical inclusion criteria (STITCH combined score ≥ 0.400, supplemented by literature curation). A total of 10 genes were shared across all three groups representing the core network for hepatic homeostasis. Notably, VP demonstrated a distinct specific overlap with DILI-associated targets (n = 12) suggesting functional enrichment of mechanisms involved in acute detoxification and toxin-induced liver injury, such as bile acid regulation and oxidative stress defense. Numbers indicate the gene counts within each intersection. A comprehensive list of genes for each intersection is provided in Table S3.

To evaluate the therapeutic positioning of VP within the broader landscape of liver injury management, its pharmacological target profile was comparatively analyzed against conventional HP group and established DILI-associated targets. Using identical analytical criteria (STITCH combined score ≥ 0.400), VP yielded 71 curated targets, while the HP group generated 57 consensus targets. For DILI-associated targets, an initial pool of 1464 genes was retrieved through integrated database mining (using terms such as “drug-induced liver injury,” “hepatotoxicity,” and “toxic liver injury”). To ensure high functional relevance and minimize false positives, this pool was strictly filtered to include only 109 high-confidence targets with relevance scores ≥ 5. The numerical consistency of each target set and intersection size was manually verified to ensure consistency with the Venn diagram output (Figure 1, Table S3). The target sets were compared, revealing distinct functional clusters. The curated VP target set (n = 71), as defined in Section 3.1.2, was compared with targets from the conventional HP group and DILI-associated genes to characterize its unique therapeutic signature.

(1) Shared Core Targets (VP ∩ HP ∩ DILI).

Ten targets were common to all the three groups: ABCB1, CASP3, CYP1A1, CYP1A2, CYP1B1, CYP2C8, CYP3A4, IL6, MAPK8, and SIRT1. These genes represent a shared pharmacological foundation encompassing inflammatory regulation (IL6), apoptosis control (CASP3 and MAPK8), and cellular stress adaptation (SIRT1). Notably, the prominent presence of multiple cytochrome P450 enzymes—key mediators in phase I drug metabolism—suggests that both VP and conventional formulae converge on maintaining hepatic metabolic homeostasis and detoxification efficiency [13,21,37].

(2) VP–DILI Specific Targets (VP ∩ DILI\HP)

Importantly, 12 targets were uniquely shared between VP and DILI but were absent in the HP group. These included regulators of bile acid transport (ABCB11 and CYP7A1) [21,25], antioxidant defense (NFE2L2 and HMOX1) [15,20], and critical signaling nodes for hepatic recovery (BCL2, STAT3, TNF, EGFR, TGFB1, UGT1A1, PPARA, and PPARG) [19,20,22]. The simultaneous enrichment of hepatobiliary transport and stress response pathways suggested that VP possessed a coordinated adaptive module specifically tailored to acute hepatotoxic stress, rather than general metabolic support.

(3) VP-Exclusive Targets

VP identified 32 exclusive targets that were not shared by either the HP or DILI groups including PRKN, PINK1, BAX, FOXO3, GPBAR1, NR0B2, and SLC10A2. These targets were highly enriched for coordinated processes involving mitochondrial quality control via mitophagy regulation (PINK1 and PRKN), the mitochondrial apoptosis pathway (BAX), and adaptive bile acid signaling (GPBAR1 and NR0B2) [10,11,19,37]. Specifically, the inclusion of FOXO3 and GPBAR1 suggests that VP may exert unique hepatoprotective effects by enhancing cellular stress resistance and modulating non-canonical bile acid pathways that are underrepresented in conventional formulas [16,20]. Rather than representing isolated pharmacological effects, these VP-specific nodes contribute to a broader integrated response that may be associated with recovery under acute and complex hepatic stress.

(4) VP–HP Specific targets

Additionally, 17 targets were exclusively shared between the VP and HP groups, including AKT1, AR, CCL2, ESR1, MMP9, and TP53. These targets are associated with general cellular signaling, tissue repair, and chronic stress responses [13,23], reflecting the shared lineage of VP with traditional hepatoprotective frameworks.

(5) HP–DILI Shared targets

In contrast, only four targets were shared between the HP and DILI groups (GSTP1, HIF1A, MTHFR, and PTGS2). These genes were enriched in the pathways associated with chronic inflammation and angiogenesis. This suggests that conventional formulas may preferentially influence long-term inflammatory adaptation, whereas VP is more directly associated with acute detoxification and mitochondrial recovery [33,34].

Collectively, these findings indicated that although VP shared a foundational network with conventional formulas, its target profile demonstrated a significantly stronger and more specific enrichment of DILI-associated pathological mechanisms [3,4,13]. Specifically, the integration of both predicted and evidence-based targets enabled VP to selectively modulate interconnected pathways involving oxidative stress regulation, bile acid homeostasis, and mitochondrial quality control [21,22,37]. This preferential alignment provided a robust mechanistic basis for the disease-focused analyses presented in subsequent sections.

#### 3.2.2. Functional Interpretation of DILI-Aligned Targets

To clarify the biological implications of these target clusters, functional enrichment analysis was performed on the 22-target module, consisting of the shared core and VP–DILI-specific clusters, representing the mechanistic interface between VP pharmacological targets and DILI-associated pathology. GO biological process enrichment revealed strong enrichment in pathways related to xenobiotic metabolism, oxidative stress response, inflammatory signaling, and apoptosis regulation. In particular, xenobiotic metabolic processes and responses to xenobiotic stimuli were significantly enriched, reflecting the involvement of multiple drug-metabolizing enzymes including CYP1A1, CYP1A2, CYP1B1, CYP2C8, CYP3A4, and UGT1A1 and transporters such as ABCB1 and ABCB11, which collectively participate in hepatic detoxification and xenobiotic clearance [13,22]. Additional enriched processes included response to oxidative stress, response to hypoxia, and positive regulation of apoptotic processes involving key regulatory genes such as NFE2L2, HMOX1, SIRT1, STAT3, CASP3, and BCL2. These stress-responsive pathways represent critical adaptive mechanisms that determine hepatocyte survival following toxin-induced injury.

Consistent with these GO findings, KEGG pathway analysis highlighted several liver injury-relevant signaling pathways including lipid and atherosclerosis, non-alcoholic fatty liver disease, and alcoholic liver disease, demonstrating a convergence of metabolic stress and inflammatory signaling in hepatic pathology. Notably, enrichment of the bile secretion pathway involving ABCB11, UGT1A1, CYP3A4, and CYP7A1, indicated a potential role of VP targets in regulating hepatobiliary transport and bile acid homeostasis, which are critical factors in preventing cholestatic liver injury [17,22,37]. Furthermore, stress-responsive signaling pathways such as AGE–RAGE, TNF, HIF-1, and MAPK were also significantly enriched. These pathways integrate inflammatory signaling, oxidative stress responses, and cellular survival mechanisms, suggesting that VP modulates a coordinated regulatory network that supports detoxification, stress adaptation, and hepatocyte survival during DILI.

Taken together, these enrichment patterns indicated that the pharmacological targets of VP converged on interconnected biological modules involving xenobiotic metabolism, bile acid homeostasis, oxidative stress regulation, inflammatory signaling, and apoptosis control, thereby providing a mechanistic framework linking VP-associated targets to the molecular processes involved in DILI.

### 3.3. Physicochemical and ADME Characteristics of the VP Library

**Figure 2:**
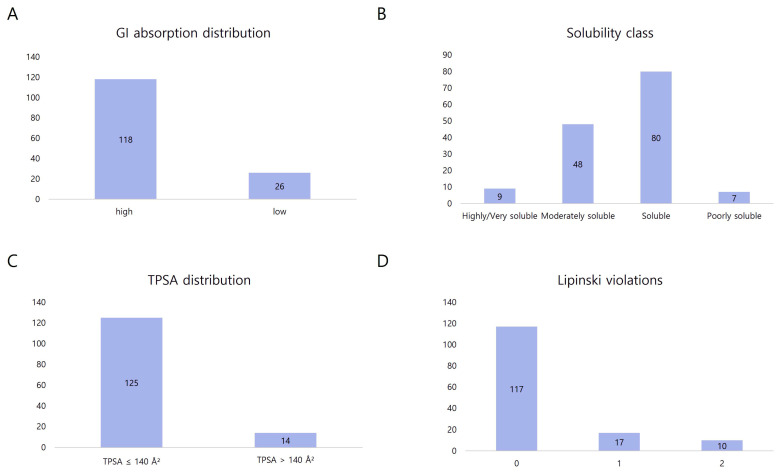
Physicochemical and ADME profiling of V-pharmacopuncture constituents. (**A**) Predicted gastrointestinal absorption distribution. (**B**) Solubility classification based on the ESOL model. (**C**) TPSA distribution indicating membrane permeability potential. (**D**) Lipinski rule violation profile representing compound drug-likeness.

To evaluate the drug-likeness and pharmacokinetic plausibility of VP, physicochemical profiling was performed on the ADME-analyzable subset of compounds. Although the standardized network pharmacology dataset comprised 133 compounds (Section 3.1), ADME screening was conducted for a broader set of 144 compounds for which canonical SMILES structures were available. This approach allowed for a comprehensive characterization of the physicochemical properties of the VP constituents. Because compound identification, ADME profiling, and target prediction rely on partially independent data sources, the compound sets used in each analytical step were not identical (Figure 2).

(1) Gastrointestinal absorption and solubility

Prediction of GI absorption indicated that 81.9% (118/144) of the compounds exhibited high absorption potential, whereas 18.1% (26/144) exhibited low absorption. The solubility analysis based on the ESOL model [26] showed that most constituents were distributed within favorable solubility ranges. Highly/Very soluble: 9 compounds (6.2%); soluble: 80 compounds (55.6%); moderately soluble: 48 compounds (33.3%); poorly soluble: 7 compounds (4.9%). Overall, 94.9% of the compounds were categorized as moderately soluble or better, suggesting adequate dissolution characteristics for biological interactions. The presence of a small fraction of poorly soluble compounds was consistent with the clinical rationale for pharmacopuncture, in which injectable delivery may overcome dissolution-related limitations associated with oral administration [14,15].

(2) Membrane permeability (TPSA distribution)

TPSA analysis, an established indicator of membrane permeability [26], demonstrated that 86.8% (125/144) of compounds possessed TPSA values ≤ 140 Å^2^, consistent with favorable cellular permeability. Only 13.2% (19/144) of the compounds exceeded this threshold. These findings suggested that most VP constituents were structurally capable of penetrating cellular membranes and interacting with intracellular molecular targets implicated in liver injury.

(3) Drug-likeness evaluation (Lipinski’s Rule of Five)

The drug-likeness assessment revealed strong compliance with Lipinski’s Rule of Five [26], with 117 compounds (81.3%) showing no violations, 17 (11.8%) showing one violation, and 10 (6.9%) showing two or more violations. The predominance of compounds satisfying these classical drug-likeness criteria indicates that the VP constituents largely occupy chemically favorable pharmacological spaces, supporting their predicted capacity for protein target engagement in subsequent network analyses.

Collectively, ADME profiling provided physicochemical characterization of the VP constituent library. Because VP is administered via pharmacopuncture rather than oral ingestion, these parameters should not be interpreted as pharmacokinetic evidence for the injectable formulation. Rather, they serve two complementary purposes: (1) as a general indicator of drug-likeness and molecular accessibility of the constituent compounds, and (2) to contextualize the rationale for the pharmacopuncture delivery route. Notably, a subset of compounds exhibited physicochemical characteristics that would limit oral bioavailability—including 4.9% classified as poorly soluble and 13.2% with TPSA exceeding 140 Å^2^. Such compounds may benefit from direct injectable delivery, which bypasses gastrointestinal absorption barriers and first-pass metabolism. In this context, the physicochemical data are consistent with the pharmacopuncture delivery rationale rather than predicting oral pharmacokinetics.

The presence of constituents with diverse physicochemical properties, including those with limited aqueous solubility, was consistent with an integrated extraction process that combined ethanol extraction and aqueous distillation. This formulation strategy allowed inclusion of both hydrophilic and lipophilic bioactive constituents in injectable preparations.

### 3.4. Herb–Compound–Target Interaction Network Analysis

**Figure 3:**
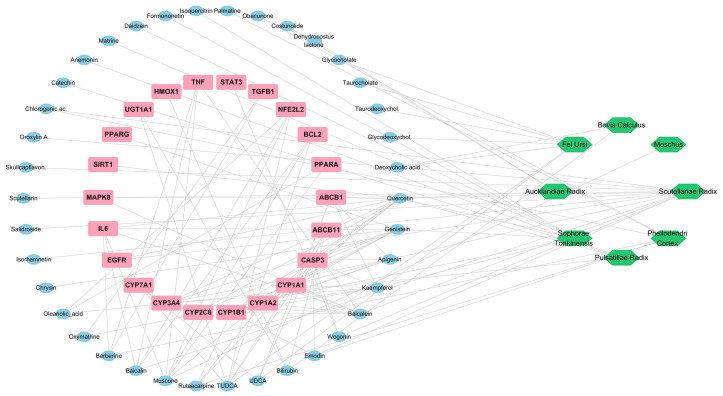
Herb–compound–target interaction network of V-pharmacopuncture. The network illustrates the interactions among 8 herbal components (green nodes), bioactive compounds (blue nodes), and 22 liver injury-associated molecular targets (pink nodes). Targets were selected from a curated 71-target dataset based on overlap with drug-induced liver injury (DILI)-related pathways to enhance mechanistic interpretability. Topological analysis revealed a central inflammatory regulatory cluster composed of TNF, IL6, STAT3, and CASP3, representing key signaling nodes involved in inflammatory amplification and hepatocyte survival responses during toxic stress. Additional targets linked to oxidative stress regulation (NFE2L2, HMOX1) and hepatobiliary transport (ABCB11) were integrated within the network, highlighting the multilayered regulatory architecture associated with xenobiotic detoxification and cellular stress adaptation. The network structure demonstrated that multiple VP-derived compounds simultaneously interacted with several targets, consistent with a multi-component and multi-target pharmacological mechanism underlying VP-mediated hepatoprotection.

To elucidate the system-level pharmacological mechanisms underlying VP, a herb–compound–target (H–C–T) interaction network was constructed using Cytoscape [30]. This network integrated VP-derived bioactive compounds with liver injury-associated molecular targets identified through comparative analysis of DILI datasets. As described in Section 3.1, an initial pharmacological landscape comprising 133 compounds and 71 curated targets was established. While this full dataset enabled comprehensive mapping of compound–target interactions, downstream mechanistic interpretation required the prioritization of disease-relevant targets. Therefore, a representative subset of 22 targets that overlapped with DILI-associated pathways was selected for network visualization and subsequent topological analysis. The resulting H–C–T network consisted of 8 herb nodes representing the constituent materials of VP, bioactive compound nodes derived from TCMSP screening and literature-supported pharmacological markers, and 22 liver injury-associated target nodes representing convergent molecular processes implicated in hepatocellular stress and recovery (Figure 3).

The network demonstrated a densely interconnected architecture rather than isolated compound–target relationships, indicating coordinated regulation across multiple biological pathways. Topological analysis revealed that several inflammatory and stress response regulators, including TNF, IL6, STAT3, and CASP3, formed a highly connected central cluster within the network, representing the key regulatory hubs involved in inflammatory signaling and hepatocyte survival during toxic stress. In addition to this inflammatory core, other functionally relevant nodes—including regulators of oxidative stress defense (NFE2L2 and HMOX1) and hepatobiliary transport (ABCB11)—were connected to the network, reflecting cellular responses to xenobiotic injury and bile acid homeostasis. Importantly, representative VP constituents, such as muscone, TUDCA, baicalin, and berberine, were simultaneously connected to multiple functional targets, suggesting cross-regulatory roles linking inflammatory modulation, oxidative stress adaptation, and metabolic detoxification. Overall, the network topology suggests that VP may act through multiple interconnected stress-response systems rather than a single dominant pathway, providing a plausible mechanistic framework for the subsequent analyses (Figure 3).

### 3.5. PPI Network Analysis and Hub Gene Identification of VP–DILI Overlapping Targets

**Figure 4:**
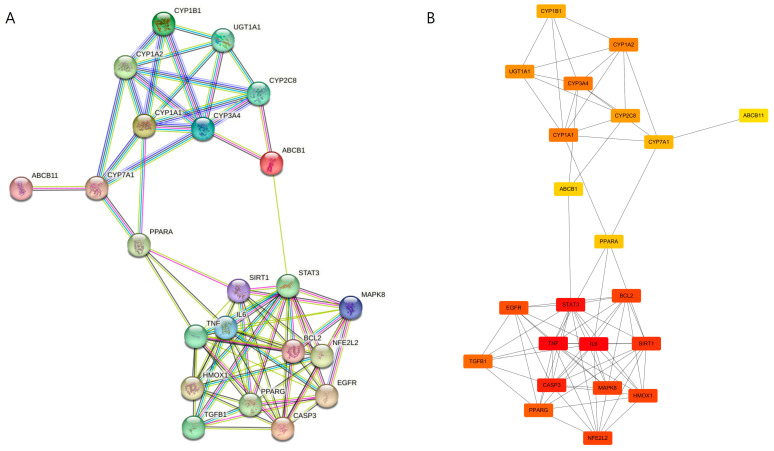
PPI network analysis of 22 VP–DILI overlapping targets. (**A**) STRING database visualization showing two functional clusters: a xenobiotic/metabolic module (upper; CYP1A1, CYP1A2, CYP1B1, CYP3A4, CYP2C8, UGT1A1, CYP7A1, ABCB1, ABCB11, PPARA) and an inflammatory/stress-response core (lower; TNF, IL6, STAT3, CASP3, NFE2L2, HMOX1, BCL2, MAPK8, SIRT1, EGFR, PPARG, TGFB1). Edges indicate high-confidence protein interactions (confidence score ≥ 0.700). (**B**) Topological hub analysis using the cytoHubba MCC algorithm. Nodes are colored by MCC score (red: highest; yellow: lowest). IL6 and TNF ranked as the top hub genes (score: 4566), followed by STAT3 (4561) and CASP3 (3720), identifying the central inflammatory regulatory axis of VP–DILI interactions.

To identify the central pharmacological nodes within the VP–DILI overlapping network, a PPI network was constructed using the 22 overlapping targets via the STRING database (confidence score ≥ 0.700) and imported into Cytoscape for topological analysis. The resulting network exhibited two functionally distinct clusters: an upper xenobiotic and metabolic module comprising cytochrome P450 enzymes (CYP1A1, CYP1A2, CYP1B1, CYP3A4, CYP2C8), drug transporters (ABCB1, ABCB11), and bile acid regulators (CYP7A1, PPARA); and a lower inflammatory and stress-response core comprising TNF, IL6, STAT3, CASP3, NFE2L2, HMOX1, BCL2, MAPK8, SIRT1, EGFR, PPARG, and TGFB1. The two modules were bridged primarily through PPARA and ABCB1, suggesting a coordinated link between xenobiotic metabolism and inflammatory regulation (Figure 4A).

Topological hub analysis using the MCC algorithm via the cytoHubba plugin identified IL6 and TNF as the top-ranked hub genes (score: 4566), followed by STAT3 (score: 4561) and CASP3 (score: 3720), indicating that VP’s primary interface with DILI pathology centers on modulation of the JAK/STAT3 and TNF-α signaling axes. High MCC scores were additionally observed for SIRT1 (rank 5), HMOX1 and NFE2L2 (rank 6, score: 2160), and BCL2 (rank 6), suggesting concurrent regulation of antioxidant defense (Nrf2 pathway) and apoptosis. The cytochrome P450 enzymes (CYP1A1, CYP3A4, CYP1A2), while ranked lower by MCC, were retained as key nodes representing phase I drug metabolism relevant to DILI initiation. Based on these findings, IL6, TNF, STAT3, and CASP3 were selected as core hub genes representing the central regulatory axis for further mechanistic analysis in the focused nine-gene module described in Section 3.7 (Figure 4B).

### 3.6. Functional Enrichment Analysis of VP-DILI Overlapping Targets

To investigate the biological functions and pathways associated with the 22 overlapping targets between VP and DILI, GO biological process enrichment and KEGG pathway analyses were performed using DAVID database [38]. Using a stringent FDR threshold of 0.05, multiple biological processes and signaling pathways relevant to xenobiotic metabolism, oxidative stress response, inflammatory signaling, and hepatocyte survival were significantly enriched (Table S4).

#### 3.6.1. GO Biological Process Enrichment

**Figure 5:**
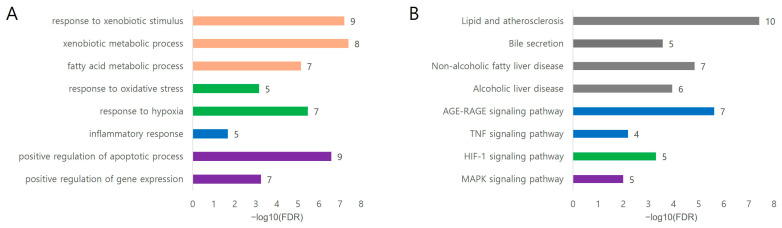
Functional enrichment analysis of VP–DILI overlapping targets. Functional enrichment analysis was performed using 22 overlapping targets between VP and drug-induced liver injury (DILI) to identify potential biological mechanisms associated with VP. (**A**) Gene Ontology (GO) biological process enrichment analysis of biological processes. Enriched GO terms were categorized into four functional modules: xenobiotic/metabolic response (orange), oxidative stress and hypoxia response (green), inflammatory response (blue), and apoptosis regulation (purple). Collectively, these processes suggest a functional transition from xenobiotic sensing and metabolic detoxification to cellular stress adaptation and survival. (**B**) KEGG pathway enrichment analysis. Significant pathways are grouped into metabolic and liver-related pathways (grey), inflammatory signaling pathways (blue), and intracellular stress-response pathways (green/purple). The analysis highlights pathways related to metabolic stress, hepatobiliary transport, and inflammatory signaling, including lipid and atherosclerosis, bile secretion, and TNF signaling. Bar length represents statistical significance as −log10(FDR), and the numbers indicate the gene count associated with each term.

GO biological process enrichment analysis revealed that overlapping targets were predominantly involved in processes associated with xenobiotic metabolism and cellular responses to toxic stimuli (Figure 5A). Among the enriched terms, the xenobiotic metabolic process and response to xenobiotic stimuli showed the highest statistical significance, indicating that the shared targets between VP and DILI were strongly associated with metabolic processing and cellular adaptation to exogenous toxic compounds. These processes involved several cytochrome P450 enzymes and transporters, including CYP1A1, CYP1A2, CYP1B1, CYP3A4, UGT1A1, ABCB1, and ABCB11, which are known to promote hepatic detoxification, drug metabolism, and fatty acid homeostasis. In addition, the enrichment of fatty acid metabolic processes and cellular stress adaptation—including responses to oxidative stress and hypoxia—further suggested the involvement of metabolic regulatory pathways and antioxidant defense systems governed by genes such as NFE2L2, HMOX1, SIRT1, STAT3, and PPARA. Furthermore, enrichment of the positive regulation of apoptotic processes and inflammatory responses indicated that the identified targets participated in key signaling pathways involving IL6, TNF, and STAT3, alongside apoptosis-related genes such as CASP3 and BCL2 [13,21]. Overall, these GO enrichment results suggest that the VP-related target network is functionally enriched in biological modules ranging from toxin sensing and metabolic detoxification to inflammatory signaling and apoptosis modulation, all of which represent key mechanisms underlying DILI pathophysiology.

#### 3.6.2. KEGG Pathway Enrichment

KEGG pathway enrichment analysis identified several signaling pathways associated with metabolic stress, inflammatory regulation, and liver disease (Figure 5B). Among these, pathways related to lipid and atherosclerosis and the AGE–RAGE signaling pathway were strongly enriched, both of which are closely associated with oxidative stress and inflammatory responses involving key signaling molecules such as IL6, MAPK8, STAT3, TNF, and BCL2. Notably, several pathways related to liver disease, including non-alcoholic fatty liver disease and alcoholic liver disease, were also significantly enriched, reflecting shared molecular mechanisms with DILI such as inflammation and hepatocyte apoptosis. In addition, the enrichment of the bile secretion pathway highlighted the involvement of hepatobiliary transport and detoxification mechanisms—mediated by ABCB11, ABCB1, UGT1A1, CYP3A4, and CYP7A1—suggesting a potential role for VP in modulating hepatobiliary homeostasis relevant to cholestatic liver injury [22,37]. Furthermore, signaling pathways such as TNF, MAPK, and HIF-1 were identified, indicating that VP-associated targets may participate in multiple stress-responsive cascades that regulate cellular adaptation to toxic or hypoxic conditions. Taken together, these KEGG findings indicate that the overlapping targets between VP and DILI are primarily associated with pathways involved in metabolic regulation, inflammatory signaling, and hepatobiliary function, thereby providing a coherent mechanistic framework into the potential hepatoprotective actions of VP.

### 3.7. Ephedra-Specific Mechanistic Analysis Based on the Focused Gene Module

#### 3.7.1. Functional Enrichment Analysis of the Ephedra-Focused Module

To refine system-level findings regarding Ephedra-associated hepatotoxicity, a focused gene module was constructed using a structured three-layer selection strategy integrating disease topology, toxin-specific mechanisms, and treatment relevance. First, four core DILI hub genes (IL6, TNF, STAT3, and CASP3) were selected according to the maximal clique centrality (MCC) ranking within the PPI network derived from VP–DILI overlapping targets. These genes represent central regulators of inflammatory signaling and hepatocyte survival and were repeatedly enriched in GO and KEGG analyses. Second, two genes (PINK1 and PRKN) were incorporated to capture mitochondrial toxicity mechanisms specifically associated with *Ephedra* exposure [10,11]. Experimental studies have shown that Ephedra alkaloids induce oxidative stress-mediated mitochondrial dysfunction and activate mitophagy pathways, supporting the biological relevance of this axis in Ephedra-related hepatotoxicity. Third, three genes (ABCB11, NFE2L2, and HMOX1) were included based on the overlap between adverse outcome pathway-associated HILI/DILI targets and VP-associated targets. These genes represent adaptive systems that govern bile acid transport, antioxidant defense, and hepatic metabolic regulation [21,22,37]. This integrative strategy resulted in a nine-gene mechanistic module linking DILI inflammatory hubs, Ephedra-specific mitochondrial stress responses, and VP-responsive hepatoprotective pathways. The nine-gene module is visually contextualized within the full 22-target PPI network (Figure 4), from which the hub genes were originally derived based on topological importance.

#### 3.7.2. PPI Network Characteristics of the Ephedra-Focused Module

**Figure 6:**
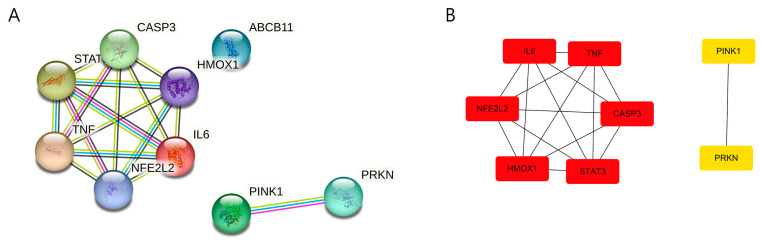
PPI network architecture of the Ephedra-focused nine-gene module. (**A**) STRING database visualization: Nodes represent proteins, and edges indicate functional associations (confidence score ≥ 0.7). The network reveals a major cluster of inflammatory/stress genes and a distinct functional pair of PINK1–PRKN. (**B**) Topological analysis (cytoHubba): Nodes are ranked and grouped by connectivity. The red cluster indicates high-degree hub genes (TNF, IL6, STAT3, CASP3, NFE2L2, HMOX1) governing systemic responses, while the yellow cluster identifies the PINK1–PRKN axis as a specific regulatory module for mitochondrial integrity.

PPI analysis of the nine-gene module revealed a highly structured functional architecture, characterized by significant interconnectivity (PPI enrichment *p*-value < 1.0 × 10^−16^), indicating that these targets do not act in isolation but through coordinated biological circuits (Figure 6, Table S5). Within this network, TNF, IL6, STAT3, and CASP3 formed a dense central cluster—the inflammatory and survival core (Red hubs in Figure 6B)—representing the primary signaling axis for inflammatory amplification and hepatocyte survival in response to toxic stress. Closely integrated into this core are NFE2L2 (Nrf2) and HMOX1, which constitute the adaptive stress and detoxification axis, suggesting that antioxidant defense systems operate alongside inflammatory regulation in the network architecture. Notably, PINK1 and PRKN formed a distinct specialized subnetwork (Yellow hubs in Figure 6A,B), highlighting a mitochondrial quality control axis associated with Ephedra-related hepatotoxicity. The relative specialization of this binary axis within the network underscores the potential role of VP in resolving mitochondrial dysfunction and restoring cellular homeostasis, distinct from generalized inflammatory pathways. Furthermore, ABCB11 serves as a functional effector linked to hepatic metabolic adaptation, facilitating the excretion of bile acids and xenobiotics, and potentially contributing to hepatic recovery.

#### 3.7.3. Functional Enrichment Analysis of the Focused Nine-Gene Module

**Figure 7:**
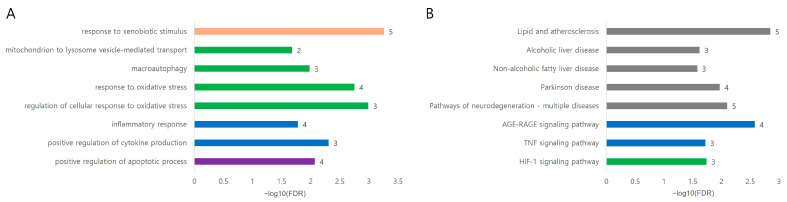
Functional enrichment analysis of the focused nine-gene module associated with VP-mediated hepatoprotection. Functional enrichment analysis was performed using nine representative targets (IL6, TNF-, STAT3, CASP3, PINK1, PRKN, NFE2L2, HMOX1, and ABCB11) derived from the VP–DILI interaction network. (**A**) Gene Ontology (GO) biological process enrichment. The enriched processes were grouped into four functional modules: xenobiotic/toxic response (orange), mitochondrial quality control, oxidative stress regulation (green), inflammatory signaling (blue), and apoptosis regulation (purple). These results illustrate a coordinated biological cascade linking toxin sensing to mitochondrial protection and inflammatory regulation. (**B**) KEGG pathway enrichment analysis. The enriched pathways highlighted liver disease-associated mechanisms (gray), inflammatory signaling pathways (blue), and cellular stress adaptation pathways (green/purple). Notably, mitochondrial quality-control-related pathways, such as Parkinson’s disease and neurodegeneration pathways, reflect the involvement of the PINK1–PRKN mitophagy axis, suggesting a potential role of mitochondrial protection in the hepatoprotective effects of VP. Bar length represents statistical significance expressed as −log10(FDR), and numbers indicate the gene counts associated with each term.

To further investigate the core biological mechanisms potentially linking VP with Ephedra-associated hepatotoxicity, functional enrichment analysis was conducted using a focused nine-gene module consisting of IL6, TNF, STAT3, CASP3, PINK1, PRKN, NFE2L2, HMOX1, and ABCB11. These genes represented key regulatory nodes identified in the VP–DILI overlapping network and additional mechanism-based targets associated with mitochondrial toxicity and hepatocellular stress responses. GO biological process enrichment analysis revealed significant enrichment in pathways related to xenobiotic response, mitochondrial quality control, oxidative stress regulation, inflammatory signaling, and apoptosis modulation (Figure 7). Among these processes, responses to xenobiotic stimuli and oxidative stress were prominently enriched, reflecting the involvement of cellular defense mechanisms activated in response to hepatotoxic compounds. Notably, mitochondrion-to-lysosome vesicle-mediated transport and macroautophagy were also significantly enriched, highlighting the role of mitophagy-related pathways mediated by the PINK1–PRKN axis [10,11]. These findings suggest that mitochondrial quality control mechanisms provide an important protective response against toxin-induced hepatocellular injury. Furthermore, inflammatory and apoptotic regulatory processes—including the inflammatory response and positive regulation of cytokine production—were primarily driven by key inflammatory mediators such as IL6, TNF, and STAT3 together with the apoptosis-related effector CASP3, suggesting a coordinated regulation of inflammatory signaling and hepatocyte survival pathways.

The KEGG pathway enrichment analysis was consistent with these observations, identifying several pathways associated with metabolic stress, inflammatory signaling, mitochondrial dysfunction, and liver disease (Figure 7). Among these, AGE–RAGE, TNF, and HIF-1 signaling pathways were significantly enriched, indicating the activation of stress-responsive signaling networks to regulate inflammatory responses and cellular adaptation to toxic insults. Importantly, multiple liver disease–related pathways including alcoholic liver disease and non-alcoholic fatty liver disease were also enriched, suggesting that the identified targets participated in molecular mechanisms commonly associated with hepatic injury. In addition, enrichment of Parkinson’s disease and neurodegenerative pathways indicated mitochondrial quality-control pathways mediated by PINK1 and PRKN, which are well-known regulators of mitophagy and mitochondrial homeostasis, to be affected [39,40]. Collectively, these results indicated that the focused gene module captured a coordinated regulatory network linking xenobiotic stress sensing, mitochondrial quality control, oxidative stress defense, inflammatory signaling, and apoptosis regulation. These findings provide a plausible computational framework suggesting that VP may contribute to hepatic homeostasis through multi-target modulation of stress-responsive pathways, pending experimental validation.

#### 3.7.4. Clinical Contextualization (Case-Based Interpretation)

To contextualize the network-derived mechanisms, the Ephedra-focused module was interpreted along with a previously documented clinical course of suspected Ephedra-associated hepatocellular injury treated with VP. In that case, a 30-year-old female developed marked aminotransferase elevation (AST 399 IU/L; ALT 709 IU/L) following the ingestion of an herbal decoction containing ES. Causality assessment using the updated RUCAM yielded a score of +7 (“probable”) [5,41]. Liver enzyme levels normalized within 10 days of VP administration, representing a relatively rapid recovery course compared to the typical clinical observations in DILI [12,13]. Although a causal inference could not be established based on a single clinical observation, the clinical recovery pattern was qualitatively consistent with three biological axes identified in the nine-gene module: (1) inflammatory regulation (TNF–IL6–STAT3), (2) mitochondrial repair (PINK1–PRKN), and (3) bile acid adaptation (NFE2L2–HMOX1–ABCB11).

#### 3.7.5. Integrated Mechanistic Interpretation

Taken together, this focused analysis suggests that VP may be associated with three interconnected biological axes in Ephedra-associated hepatotoxicity: (1) inflammatory regulation centered on TNF–IL6–STAT3 signaling, (2) mitochondrial quality control mediated by the PINK1–PRKN mitophagy pathway, and (3) metabolic and bile acid homeostasis governed by NFE2L2 and HMOX1–ABCB11 signaling. These findings suggest that VP may act through multiple adaptive stress-response systems rather than a single dominant pathway. This multilayered regulation is consistent with the principles of network pharmacology and provides a biologically plausible framework linking molecular predictions to the observed clinical recovery. Given the exploratory nature of this integrative analysis and the absence of direct experimental validation, these findings should be considered as hypothesis-generating, providing a robust foundation for future mechanistic and clinical investigations.

## 4. Discussion

DILI is clinically challenging and is one of the leading causes of acute liver dysfunction associated with both conventional pharmaceuticals and herbal medicines [1,3,4]. Although herbal medicines have been used for centuries, the increasing global use of botanical products has led to a growing recognition of HILI [2,6]. Among herbal medicines implicated in hepatotoxicity, ES is occasionally associated with hepatocellular injury, particularly when used in high doses or in combination with other herbal preparations [8,9]. Despite these concerns, therapeutic strategies that actively promote hepatic recovery in HILI are limited, and mechanistic explanations for recovery following traditional interventions remain insufficiently explored [5,12]. In particular, the molecular mechanisms by which multi-component herbal formulations counteract toxin-induced hepatocellular injury are poorly understood.

In this study, we investigated the potential hepatoprotective mechanisms of a multicomponent pharmacopuncture formulation composed of Moschus, Fel Ursi, Calculus Bovis, Scutellariae Radix, Phellodendri Cortex, Pulsatillae Radix, Sophorae Tonkinensis Radix, and Aucklandiae Radix. By integrating network pharmacology, PPI, and functional enrichment, we explored the potential molecular mechanisms underlying the hepatoprotective effects of this pharmacopuncture formulation against Ephedra-associated liver injury. providing a mechanistic framework that links VP intervention to the molecular processes underlying DILI recovery

### 4.1. Multi-Target Pharmacological Architecture of VP

Network pharmacology analysis identified 22 overlapping VP-associated targets and DILI–related genes. These overlapping targets represent molecular interfaces where VP may interact with the pathological mechanisms involved in hepatotoxicity. Rather than acting through a single molecular target, the network structure demonstrated that VP interacted with a densely interconnected regulatory system involving inflammatory signaling, oxidative stress regulation, metabolic adaptation, and apoptosis-related pathways. To further refine the mechanistic interpretation, a focused nine-gene mechanistic module consisting of TNF, IL6, STAT3, CASP3, PINK1, PRKN, NFE2L2, HMOX1, and ABCB11, was constructed using evidence from literature. These genes collectively represent three interconnected biological processes associated with hepatocellular injury and recovery: inflammatory signaling, mitochondrial quality control, and hepatobiliary detoxification [10,11,22]. Within this module, the inflammatory mediators TNF, IL6, and STAT3 represent central regulators of acute inflammatory responses during liver injury [3,4,20]. These cytokine signaling pathways amplify hepatocellular damage by activating downstream inflammatory cascades and immune cell recruitment. CASP3, a key mediator of apoptosis, plays a central role in determining hepatocyte fate by integrating inflammatory and mitochondrial stress signals [19]. The coexistence of inflammatory regulators, mitochondrial quality control genes, and detoxification-related targets suggests that VP may be associated with coordinated modulation of multiple biological processes relevant to hepatocellular injury, rather than acting through a single dominant pathway. Such a multitarget pharmacological architecture is consistent with the complex pathophysiology of DILI [3,4,13], which typically involves overlapping processes including oxidative stress, immune activation, mitochondrial dysfunction, and hepatocyte apoptosis.

### 4.2. Mitochondrial Quality Control and Ephedra-Associated Hepatotoxicity

A distinguishing feature of Ephedra-associated hepatotoxicity is mitochondrial dysfunction and oxidative stress. Experimental studies have demonstrated that ephedrine exposure disrupts mitochondrial respiration and increases the production of reactive oxygen species (ROS), leading to hepatocyte injury. In response to mitochondrial damage, cells undergo mitophagy to selectively remove dysfunctional mitochondria and maintain cellular homeostasis [10,11]. In the present study, PPI analysis revealed that PINK1 and PRKN formed distinct functional subnetworks. These two genes are important for mitophagy, a process that selectively removes damaged mitochondria via autophagic degradation. Activation of the PINK1–PRKN pathway prevents the accumulation of dysfunctional mitochondria and limits the release of proapoptotic signals, thereby protecting hepatocytes from oxidative damage [39,40]. Functional enrichment analysis was consistent with this mechanistic axis, identifying the biological processes associated with mitochondrial quality control including mitophagy-related pathways and intracellular vesicle-mediated transport. These findings suggested that VP might contribute to the restoration of mitochondrial homeostasis during toxin-induced liver injury. TUDCA is well known for its cytoprotective properties including stabilization of mitochondrial membranes and reduction in endoplasmic reticulum stress, suggesting a plausible mechanistic link between VP constituents and the regulation of mitochondrial stress responses relevant to the PINK1–PRKN axis [19].

### 4.3. Bile Acid Transport and Metabolic Adaptation

In addition to mitochondrial dysfunction, disturbances in bile acid metabolism and hepatobiliary transport are frequently implicated in drug- and herb-induced liver injuries. Impaired bile acid excretion can lead to intracellular accumulation of toxic bile acids which further aggravates hepatocellular injury [17]. Among the targets identified in the focused module, ABCB11(also known as the bile salt export pump (BSEP)) is important for secretion of bile acid from hepatocytes in the bile canaliculi [42]. ABCB11 dysfunction is strongly associated with cholestatic liver injury and bile acid accumulation [22]. Notably, UDCA and TUDCA—key bile acid-derived constituents of VP—are known to interact with bile acid transport pathways, suggesting that VP constituents may potentially contribute to hepatobiliary homeostasis during toxin-induced liver injury, although experimental validation is required to confirm functional relevance. In addition, the antioxidant regulatory genes, NFE2L2 and HMOX1, were important for detoxification. NFE2L2 (Nrf2) is a master transcription factor that activates cellular antioxidant defense systems including HMOX1 induction [21]. Activation of the NFE2L2–HMOX1 pathway enhances cellular resistance to oxidative stress and promotes detoxification. The simultaneous involvement of antioxidant regulation and bile acid transport suggested that VP may contribute to hepatic recovery through coordinated metabolic adaptation and detoxification.

### 4.4. Contribution of Bioactive Compounds in VP

The pharmacological plausibility of VP is supported by the presence of multiple bioactive compounds with experimentally documented hepatoprotective activities. For example, muscone, a major component derived from Moschus, has anti-inflammatory and antioxidant effects and attenuates liver injury in experimental models [16,43,44]. These findings suggest that Muscone contributes to the hepatoprotective potential of VP by modulating oxidative stress–related pathways. Similarly, TUDCA, a bile acid derivative found in Fel Ursi, exhibits well-established cytoprotective properties. TUDCA stabilizes the mitochondrial membranes, alleviates endoplasmic reticulum stress, and suppresses apoptosis-related signaling pathways, thereby protecting hepatocytes from toxin-induced injury [19]. In addition, baicalin (derived from Scutellariae Radix) exhibits hepatoprotective, anti-inflammatory, and antioxidant activities in experimental models of liver injury [21]. Muscone can enhance cellular permeability and facilitate drug delivery in certain experimental systems, and thus, influence the bioavailability of coexisting compounds in multi-component formulations [45]. Furthermore, multicomponent herbal formulations exert enhanced pharmacological effects through synergistic interactions between the constituent compounds [46,47]. Taken together, the animal- and plant-derived bioactive constituents of the VP formulation might synergistically modulate interconnected biological pathways for hepatocellular injury and recovery.

### 4.5. Clinical Relevance and Biological Plausibility

The clinical case considered in this study demonstrated rapid normalization of elevated liver enzyme levels following VP treatment. In this case, the patient exhibited marked aminotransferase elevation (AST 399 IU/L; ALT 709 IU/L) following ingestion of an Ephedra-containing herbal decoction. After VP administration, the liver enzyme levels normalized within 10 days. Although a causal inference cannot be established based on a single clinical observation, the rapid biochemical recovery observed in this case is notable compared with the typical clinical course of hepatocellular DILI, which require several weeks or months for full biochemical normalization after discontinuation of the causative agent [3,12]. The network-derived molecular mechanisms identified in the present study provide a plausible explanation for these clinical observations. The simultaneous modulation of inflammatory signaling (TNF–IL6–STAT3 axis), mitochondrial quality control (PINK1–PRKN axis), and hepatobiliary detoxification (NFE2L2–HMOX1–ABCB11 axis) may be associated with coordinated cellular responses relevant to hepatocyte recovery, which is consistent with the current mechanistic understanding of DILI pathophysiology [13]. In contextualizing VP’s mechanistic profile, it is worth noting that clinically established hepatoprotective agents such as S-adenosylmethionine, essential phospholipids, alpha-lipoic acid, and thiotriazoline share mechanistic overlap with certain VP targets (particularly NFE2L2, HMOX1, and TNF/IL6); however, the co-enrichment of PINK1–PRKN-mediated mitophagy regulation alongside ABCB11-mediated bile acid transport may represent a mechanistic combination particularly relevant to the specific pathophysiology of ephedrine-induced hepatotoxicity. These observations are based on in silico target predictions and do not constitute a clinical efficacy comparison; systematic experimental comparisons remain an important direction for future research.

### 4.6. Limitations and Future Perspectives

The present study has several limitations that should be considered when interpreting its findings. First, network pharmacology analyses rely on database-derived target prediction and enrichment analyses, which may not fully capture the complexity of in vivo pharmacological interactions. The STITCH confidence threshold (≥0.400) was selected to balance network coverage and specificity, consistent with standard practice in network pharmacology; however, this threshold influences the composition of the target set, and results may differ at higher stringency thresholds. Second, the nine-gene mechanistic module was constructed through an integrative three-layer strategy combining data-driven PPI hub ranking, toxin-specific literature evidence, and disease-pathway overlap, rather than derived exclusively from unsupervised network analysis. While this approach is explicitly hypothesis-directed and reflects the clinical context of the study, the module should be interpreted as a biologically motivated framework rather than as an emergent network finding. Third, the present study did not include molecular docking or structural binding analysis. Future studies employing rigorous structure-based approaches—including binding site validation with co-crystallized reference ligands, redocking validation, and post-docking molecular dynamics simulations with binding free-energy estimation (e.g., MM-PBSA/MM-GBSA)—would provide additional structural evidence for the predicted compound–target interactions identified in this network pharmacology analysis. Fourth, this study did not include a systematic comparison of VP with clinically established hepatoprotective agents such as S-adenosylmethionine, essential phospholipids, thiotriazoline, or alpha-lipoic acid. Such comparisons would provide important context regarding the relative pharmacological positioning of VP; however, they were beyond the scope of the present mechanistic analysis and represent a valuable direction for future studies. Fifth, this study was based on a single clinical case; therefore, the therapeutic efficacy of VP cannot be generalized, and causality cannot be established. Causality assessment of DILI is complex and requires careful clinical evaluation and standardized diagnostic approaches [39]. Future studies integrating experimental validation using in vitro hepatocyte models of ephedrine-induced injury, animal models of Ephedra-associated hepatotoxicity, and larger clinical cohorts are warranted to confirm the mechanistic predictions generated by this analysis.

## 5. Conclusions

This study explored the potential hepatoprotective mechanisms of VP against Ephedra-associated liver injury using a network pharmacology approach. Our analysis suggested that VP may modulate multiple biological processes involved in hepatotoxic stress, including inflammatory signaling, oxidative stress responses, mitochondrial quality control, and hepatobiliary transport. These findings provide a plausible mechanistic framework for investigating the potential therapeutic relevance of VP in herb-induced liver injury. However, further experimental and clinical studies are warranted to validate these computational predictions.

## Figures and Tables

**Table 1:** Stepwise Identification and Prioritization of Bioactive Compounds and Pharmacological Targets for V-Pharmacopuncture.

Step	Category	Item	Number	Selection Rationale
1	Compound identification	Raw candidate pool (TCMSP)	148	Initial retrieval from botanical databases (after removing 6 duplicates)
		Literature-curated markers	10	Inclusion of animal-derived (e.g., UDCA, Muscone) and key botanical markers
		Total candidate compounds	158	Integrated pool for standardization (InChIKey obtained)
2	Physicochemical profiling	ADME-analyzable compounds	144	Compounds with retrievable SMILES structures
3	Target prediction	Mapped unique compounds	133	Compounds successfully identified and mapped in STITCH (25 unmapped excluded)
		STITCH-derived targets	57	Combined score ≥ 0.400
		Literature-supported targets	14	Experimentally validated hepatoprotective mechanisms
		Total VP targets	71	Integrated pharmacological target set
4	Disease alignment	VP–DILI overlapping targets	22	Mechanistic prioritization
5	Focused module	Ephedra-focused gene module	9	Toxin-specific mechanistic integration

Abbreviations: VP, V-pharmacopuncture; TCMSP, TCM Systems Pharmacology database; ADME, absorption/distribution/metabolism/excretion; SMILES, simplified molecular input line entry system; DILI, drug-induced liver injury; UDCA, ursodeoxycholic acid. Note: The final 71-target set integrates STITCH-predicted interactions and manually curated targets [9,10,16,18,19,20,21,24]. See Table S2 for a full list of the 158 compounds and their structural identifiers.

## Data Availability

The data presented in this study are available within the article and its Supplementary Materials. The public datasets analyzed in this study were retrieved from the TCMSP (https://tcmsp-e.com/, accessed on 9 February 2026) and GeneCards (https://www.genecards.org/, accessed on 9 February 2026) databases.

## Supplementary Materials

The following supporting information can be downloaded at https://www.mdpi.com/article/10.3390/medicina62050849/s1, Table S1: Gene target distribution across the intersection groups of the Venn diagram; Table S2: Functional enrichment analysis (GO) of the 22 core targets of VP against DILI; Table S3: KEGG pathway enrichment analysis of the 22 core targets of VP; Table S4: Herbal composition of hepatoprotective reference prescriptions used in the comparative analysis; Table S5: Comprehensive list of 158 candidate compounds in VP and their database mapping status; Table S6: Literature-supported targets manually incorporated into the V-pharmacopuncture (VP) pharmacological network (n = 14).

## Abbreviations

The following abbreviations are used in this manuscript:

DILI	Drug-induced liver injury
HILI	Herb-induced liver injury
PPI	Protein–protein interaction
GO	Gene Ontology
KEGG	Kyoto Encyclopedia of Genes and Genomes
TCMSP	Traditional Chinese Medicine Systems Pharmacology
VP	V-pharmacopuncture
ES	Ephedra sinica
UDCA	Ursodeoxycholic acid
TUDCA	Tauroursodeoxycholic acid

## References

[1] David S., Hamilton J.P. (2010). Drug-induced Liver Injury. US Gastroenterol. Hepatol. Rev..

[2] Lee W.-J., Kim H.-W., Lee H.-Y., Son C.-G. (2015). Systematic review on herb-induced liver injury in Korea. Food Chem. Toxicol..

[3] Fontana R.J., Liou I., Reuben A., Suzuki A., Fiel M.I., Lee W., Navarro V. (2023). AASLD practice guidance on drug, herbal, and dietary supplement–induced liver injury. Hepatology.

[4] European Association for the Study of the Liver (2019). EASL Clinical Practice Guidelines: Drug-induced liver injury. J. Hepatol..

[5] Kobayashi T., Iwaki M., Nogami A., Yoneda M. (2023). Epidemiology and Management of Drug-induced Liver Injury: Importance of the Updated RUCAM. J. Clin. Transl. Hepatol..

[6] Shin H.K., Huang R., Chen M. (2023). In silico modeling-based new alternative methods to predict drug and herb-induced liver injury: A review. Food Chem. Toxicol..

[7] Łupina K., Nowak A., Jabłońska A., Potaczek A., Salacha J., Ilkiewicz Ł., Kalisz A., Janczura J. (2025). Herb-Induced Liver Injury. Livers.

[8] Tang S., Ren J., Kong L., Yan G., Liu C., Han Y., Sun H., Wang X.-J. (2023). Ephedrae Herba: A Review of Its Phytochemistry, Pharmacology, Clinical Application, and Alkaloid Toxicity. Molecules.

[9] LiverTox: Clinical and Research Information on Drug-Induced Liver Injury [Internet]. Bethesda (MD): National Institute of Diabetes and Digestive and Kidney Diseases; 2012—Ephedra. [Updated 10 February 2018]. https://www.ncbi.nlm.nih.gov/books/NBK548711/.

[10] Lee A.Y., Jang Y., Hong S.H., Chang S.H., Park S., Kim S., Kang K.S., Kim J.E., Cho M.H. (2017). Ephedrine-induced mitophagy via oxidative stress in human hepatic stellate cells. J. Toxicol. Sci..

[11] Lee A.Y. (2018). Hepatotoxic Mechanisms of Ephedrine and Dihydroceramide Through Impaired Autophagy. Ph.D. Dissertation.

[12] LiverTox: Clinical and Research Information on Drug-Induced Liver Injury [Internet]. Bethesda (MD): National Institute of Diabetes and Digestive and Kidney Diseases; 2012—Clinical Course and Diagnosis of Drug Induced Liver Disease. [Updated 4 May 2019]. https://www.ncbi.nlm.nih.gov/books/NBK548733/.

[13] Ashby K., Zhuang W., González-Jimenez A., Alvarez-Alvarez I., Lucena M.I., Andrade R.J., Aithal G.P., Suzuki A., Chen M. (2021). Elevated bilirubin, alkaline phosphatase at onset, and drug metabolism are associated with prolonged recovery from DILI. J. Hepatol..

[14] Hwang J.H., Jung H.W., Jung C. (2019). Evaluation of the Single-Dose Toxicity of TA Pharmacopuncture in Rats. J. Pharmacopunct..

[15] Hwang J.H., Jaseung K., Chul J. (2020). Evaluation of the Single-Dose Toxicity of Capsaicin Pharmacopuncture in Rats. J. Acupunct. Res..

[16] Lee K.-J., Yoon H.-C., Lee J.-S., Kwon K.-R. (2005). A Literary Study on Moschus. J. Pharmacopunct..

[17] Beuers U., Trauner M., Jansen P., Poupon R. (2015). New paradigms in the treatment of hepatic cholestasis: From UDCA to FXR, PXR and beyond. J. Hepatol..

[18] Kong B., Wang L., Chiang J.Y., Zhang Y., Klaassen C.D., Guo G.L. (2012). Mechanism of tissue-specific farnesoid X receptor in suppressing the expression of genes in bile-acid synthesis in mice. Hepatology.

[19] Rodrigues C.M., Sola S., Nan Z., Castro R.E., Ribeiro P.S., Low W.C., Steer C.J. (2003). Tauroursodeoxycholic acid reduces apoptosis and protects against neurological injury after acute hemorrhagic stroke in rats. Proc. Natl. Acad. Sci. USA.

[20] Kong W.J., Wei J., Zuo Z.Y., Wang Y.M., Song D.Q., You X.F., Zhao L.X., Pan H.N., Jiang J.D. (2008). Combination of simvastatin with berberine improves the lipid-lowering efficacy. Metabolism.

[21] Yang J.Y., Li M., Zhang C.L., Liu D. (2021). Pharmacological properties of baicalin on liver diseases: A narrative review. Pharmacol. Rep..

[22] Garzel B., Yang H., Zhang L., Huang S.M., Polli J.E., Wang H. (2014). The role of bile salt export pump gene repression in drug-induced cholestatic liver toxicity. Drug Metab. Dispos..

[23] Li X., Wei S., Niu S., Ma X., Li H., Jing M., Zhao Y. (2022). Network pharmacology prediction and molecular docking-based strategy to explore the potential mechanism of Huanglian Jiedu Decoction against sepsis. Comput. Biol. Med..

[24] Ru J., Li P., Wang J., Zhou W., Li B., Huang C., Li P., Guo Z., Tao W., Yang Y. (2014). TCMSP: A database of systems pharmacology for drug discovery from herbal medicines. J. Cheminform..

[25] Kim S., Chen J., Cheng T., Gindulyte A., He J., He S., Li Q., Shoemaker B.A., Thiessen P.A., Yu B. (2023). PubChem 2023 update. Nucleic Acids Res..

[26] Daina A., Michielin O., Zoete V. (2017). SwissADME: A free web tool to evaluate pharmacokinetics, drug-likeness and medicinal chemistry friendliness of small molecules. Sci. Rep..

[27] Szklarczyk D., Santos A., von Mering C., Jensen L.J., Bork P., Kuhn M. (2015). STITCH 5: Augmenting protein–chemical interaction networks with tissue and affinity data. Nucleic Acids Res..

[28] Stelzer G., Rosen N., Plaschkes I., Zimmerman S., Twik M., Fishilevich S., Stein T.I., Nudel R., Lieder I., Mazor Y. (2016). The GeneCards Suite: From Gene Data Mining to Disease Genome Sequence Analyses. Curr. Protoc. Bioinform..

[29] Szklarczyk D., Gable A.L., Nastou K.C., Lyon D., Kirsch R., Pyysalo S., Doncheva N.T., Legeay M., Fang T., Bork P. (2020). The STRING database in 2021: Customizable protein–protein networks, and functional characterization of user-uploaded gene/measurement sets. Nucleic Acids Res..

[30] Shannon P., Markiel A., Ozier O., Baliga N.S., Wang J.T., Ramage D., Amin N., Schwikowski B., Ideker T. (2003). Cytoscape: A software environment for integrated models of biomolecular interaction networks. Genome Res..

[31] Chin C.-H., Chen S.-H., Wu H.-H., Ho C.-W., Ko M.-T., Lin C.-Y. (2014). cytoHubba: Identifying hub objects and sub-networks from complex interactome. BMC Syst. Biol..

[32] Choi H.-S., Jung T.-Y. (2004). One Case of Drug-Induced Liver Injury Treated with Saenggangeonbi-tang. J. Intern. Korean Med..

[33] Seo H.S., Kim H.G., Joo H., Kwon J., Cho J.H. (2025). Injinoryeong-San attenuates metabolic dysfunction-associated steatohepatitis via regulation of YAP/TAZ-signaling pathway. J. Ethnopharmacol..

[34] Park J., Kim H., Lee I.S., Kim K.H., Kim Y., Na Y.C., Lee J.H., Jang H.J. (2017). The therapeutic effects of Yongdamsagan-tang on autoimmune hepatitis models. Biomed. Pharmacother..

[35] Lv S., Lei Z., Yan G., Shah S.A., Ahmed S., Sun T. (2021). Chemical compositions and pharmacological activities of natural musk (Moschus) and artificial musk: A review. J. Ethnopharmacol..

[36] Chu Z., Chen Y., Xie D., Song C., Yang L., Qin T., Zhai Z., Cao Z., Xu Y., Sun T. (2025). Ethanol extract of Moschus attenuates glutamate-induced cytotoxicity in HT22 cells by regulating the Nrf2 and MAPK pathways. J. Ethnopharmacol..

[37] Liu Y., Binz J., Numerick M.J., Dennis S., Luo G., Desai B., MacKenzie K.I., Mansfield T.A., Kliewer S.A., Goodwin B. (2003). Hepatoprotection by the farnesoid X receptor agonist GW4064 in rat models of intra- and extrahepatic cholestasis. J. Clin. Investig..

[38] Sherman B.T., Hao M., Qiu J., Jiao X., Baseler M.W., Lane H.C., Imamichi T., Chang W. (2022). DAVID: A web server for functional enrichment analysis and functional annotation of gene lists (2021 update). Nucleic Acids Res..

[39] Narendra D.P., Youle R.J. (2024). The role of PINK1–Parkin in mitochondrial quality control. Nat. Cell Biol..

[40] Pickrell A.M., Youle R.J. (2015). The roles of PINK1, parkin, and mitochondrial fidelity in Parkinson’s disease. Neuron.

[41] Danan G., Teschke R. (2015). RUCAM in Drug and Herb Induced Liver Injury: The Update. Int. J. Mol. Sci..

[42] Sohail M.I., Dönmez-Cakil Y., Szöllősi D., Stockner T., Chiba P. (2021). The Bile Salt Export Pump: Molecular Structure, Study Models and Small-Molecule Drugs for the Treatment of Inherited BSEP Deficiencies. Int. J. Mol. Sci..

[43] Wang J., Zhao X., Chen J., Liu Y., Guo Z., Lan Y., Wu Q. (2021). Investigation of muscone as transdermal penetration enhancer: Enhancing activity and molecular mechanisms. J. Drug Deliv. Sci. Technol..

[44] Wang J., Xing H., Qin X., Ren Q., Yang J., Li L. (2020). Pharmacological effects and mechanisms of muscone. J. Ethnopharmacol..

[45] Qi N., Duan W., Gao D., Ma N., Zhang J., Feng J., Li A. (2023). “Guide” of muscone modification enhanced brain-targeting efficacy and anti-glioma effect of lactoferrin modified DTX liposomes. Bioeng. Transl. Med..

[46] Zhou X., Seto S.W., Chang D., Kiat H., Razmovski-Naumovski V., Chan K., Bensoussan A. (2016). Synergistic Effects of Chinese Herbal Medicine: A Comprehensive Review of Methodology and Current Research. Front. Pharmacol..

[47] Hong M., Li S., Tan H.Y., Cheung F., Wang N., Huang J., Feng Y. (2017). A Network-Based Pharmacology Study of the Herb-Induced Liver Injury Potential of Traditional Hepatoprotective Chinese Herbal Medicines. Molecules.

