# Supplementary material for: Network Pharmacology Analysis Reveals Multi-Target Hepatoprotective Mechanisms of a Multi-Component Pharmacopuncture Against Ephedra-Associated Liver Injury with Implications for Mitochondrial Quality Control
Source: Medicina (Kaunas). 2026 Apr 29;62(5):849. doi: 10.3390/medicina62050849 (PMC13208707; doi:10.3390/medicina62050849)
Supplement: Supplementary file 1 [file medicina-62-00849-s001.zip › medicina-4251411-supplementary.pdf]

Table S1. Gene target distribution across the intersection groups of the Venn diagram

Intersection Group	Gene Count	Gene List
Shared Core (VP ∩ HP ∩ DILI)	10	ABCB1, CASP3, CYP1A1, CYP1A2, CYP1B1, CYP2C8, CYP3A4, IL6, MAPK8, SIRT1
VP–HP Specific (VP ∩ HP) - Core	17	AKT1, ALOX12, AR, ATP5A1, ATP5B, ATP5C1, CCL2, CSNK2A1, ESR1, HCK, HIBCH, MCL1, MMP9, PIM1, SLC2A2, STK17B, TP53
VP–DILI Specific (VP ∩ DILI) - Core	12	ABCB11, BCL2, CYP7A1, EGFR, HMOX1, NFE2L2, PPARA, PPARG, STAT3, TGFB1, TNF, UGT1A1
HP–DILI Specific (HP ∩ DILI) - Core	4	GSTP1, HIF1A, MTHFR, PTGS2
VP-Exclusive	32	AKR1C2, BAX, BLVRA, CEL, CFTR, CYP11B1, CYP11B2, CYP19A1, CYP21A2, ESR2, FABP6, FOXO3, GCG, GPBAR1, HMOX2, HTR2A, IL1B, MAPK1, NOS3, NR0B2, NR1H4, PNLIP, PRKN, PINK1, SLC10A2, STAT1, TAS2R31, TAS2R43, TAS2R46, TLR4, UGT1A4, UGT1A6
HP-Exclusive	26	ACTA1, ATOX1, C22orf28, CDK1, CDK2, CYP11A1, CYP2A6, DHFR, FOS, JUN, LYZ, MMP2, PARP1, PNP, PON1, RFK, SI, SLC52A3, SMPD1, SRC, TAS1R2, TAS1R3, TRPA1, TRPV1, TTPA, VEGFA

DILI-Exclusive	83	NAT2, GPT, CYP2E1, CYP2C9, ABCC2, GSTM1, GSTT1, SLC17A5, SOD2-OT1, CYP2C19, SLCO1B1, NR1I2, CYP2B6, NQO1, F2, KRT18, ALB, HLA-B, MIR7-3HG, IL4, IL10, CYP2D6, UGT2B7, POLG, CTLA4, ABCB4, HMGB1, ALPP, SOD2, CYP3A5, HLA-DQA1, GPX1, SOD1, HLA-DRB1, KRT8, GGT1, HLA-DQB1, PTPN22, PDCD1, ABCG2, HSPA5, KEAP1, ST6GAL1, DDIT3, ABCC4, APOA1, GSTA1, TPMT, LINC02605, MCTP2, ALG10B, HCP5, CYP2F1, UGT1A9, AFP, FASLG, NFKB1, CP, CERNA3, COMT, FFAR1, MT-TP, ALK, ERAP2, SLC22A12, HLA-A, ATF4, ERBB2, GPT2, XDH, OR5H8, CRP, RIPOR2, ERN1, AHR, CASP7, FABP1, CES1, HGF, HP, SEPSECS, FTCD, CDK4

Table S2. Functional enrichment analysis (GO) of the 22 core targets of VP against DILI

Category	Term	Genes	Count	P-Value	Fold Enrichment	FDR
GO BP	xenobiotic metabolic process	CYP2C8,ABCB1,UGT1A1,CYP1A2,CYP1A1,CYP1B1,ABCB11,CYP3A4	8	3.81E-11	57.22	3.61E-08
GO BP	response to xenobiotic stimulus	TGFB1,ABCB1,CASP3,STAT3,BCL2,CYP1A1,PPARG,TNF,NFE2L2	9	1.56E-10	30.70	6.36E-08
GO BP	estrogen metabolic process	CYP2C8,UGT1A1,CYP1A2,CYP1A1,CYP1B1,CYP3A4	6	2.01E-10	161.26	6.36E-08
GO BP	positive regulation of apoptotic process	IL6,MAPK8,TGFB1,CASP3,BCL2,CYP1B1,PPARG,SIRT1,TNF	9	1.11E-09	23.97	2.63E-07
GO BP	positive regulation of miRNA transcription	IL6,TGFB1,STAT3,PPARG,TNF,EGFR	6	4.95E-09	87.24	8.82E-07
GO BP	steroid metabolic process	CYP2C8,UGT1A1,CYP1A2,CYP1A1,CYP1B1,CYP3A4,CYP7A1	7	5.58E-09	44.99	8.82E-07
GO BP	xenobiotic catabolic process	CYP2C8,CYP1A2,CYP1A1,CYP1B1,CYP3A4	5	2.67E-08	147.82	3.36E-06
GO BP	response to hypoxia	TGFB1,ABCB1,CASP3,STAT3,CYP1A1,PPARA,TNF	7	2.83E-08	34.30	3.36E-06
GO BP	steroid catabolic process	CYP1A2,CYP1A1,CYP1B1,CYP3A4	4	6E-08	443.45	6.32E-06
GO BP	fatty acid metabolic process	CYP1A2,CYP1A1,CYP1B1,PPARG,ABCB11,CYP3A4,PPARA	7	7.67E-08	29.01	7.28E-06
GO BP	omega-hydroxylase P450 pathway	CYP2C8,CYP1A2,CYP1A1,CYP1B1	4	2.35E-07	295.64	2.02E-05
GO BP	retinol metabolic process	CYP2C8,CYP1A2,CYP1A1,CYP1B1,CYP3A4	5	3.03E-07	82.12	2.39E-05
GO BP	epoxygenase P450 pathway	CYP2C8,CYP1A2,CYP1A1,CYP1B1	4	1.21E-06	177.38	8.83E-05
GO BP	lipid metabolic process	CYP2C8,UGT1A1,CYP1A2,CYP1A1,CYP1B1,PPARG,CYP3A4,PPARA,CYP7A1	9	1.5E-06	9.42	0.000102
GO BP	negative regulation of miRNA transcription	TGFB1,PPARG,PPARA,TNF	4	3.86E-06	122.33	0.000244
GO BP	positive regulation of vascular endothelial growth factor production	IL6,TGFB1,STAT3,CYP1B1	4	5.22E-06	110.86	0.000309
GO BP	response to ethanol	TGFB1,STAT3,PPARA,TNF,CYP7A1	5	5.71E-06	39.59	0.000318
GO BP	long-chain fatty acid biosynthetic process	CYP2C8,CYP1A2,CYP1A1,CYP3A4	4	6.29E-06	104.34	0.00033
GO BP	olefinic compound metabolic process	CYP1A2,CYP1A1,CYP1B1	3	6.61E-06	665.18	0.00033

GO BP	response to hydrogen peroxide	CASP3,BCL2,HMOX1,SIRT1	4	0.000012	84.47	0.000569
GO BP	positive regulation of gene expression	IL6,MAPK8,TGFB1,STAT3,PPARG,TNF,NFE2L2	7	1.27E-05	12.06	0.000574
GO BP	cellular response to hypoxia	TGFB1,BCL2,PPARG,SIRT1,NFE2L2	5	1.38E-05	31.68	0.000596
GO BP	response to oxidative stress	MAPK8,BCL2,HMOX1,SIRT1,NFE2L2	5	1.72E-05	29.96	0.000709
GO BP	positive regulation of smooth muscle cell proliferation	IL6,TGFB1,HMOX1,TNF	4	0.000023	68.22	0.000886
GO BP	positive regulation of angiogenesis	STAT3,CYP1B1,HMOX1,SIRT1,NFE2L2	5	2.34E-05	27.72	0.000886
GO BP	positive regulation of transcription by RNA polymerase II	IL6,TGFB1,STAT3,PPARG,PPARA,SIRT1,TNF,EGFR,NFE2L2	9	2.55E-05	6.42	0.000903
GO BP	response to glucocorticoid	IL6,CASP3,BCL2,TNF	4	2.57E-05	65.70	0.000903
GO BP	lipid hydroxylation	CYP2C8,CYP1A1,CYP3A4	3	3.08E-05	332.59	0.00104
GO BP	negative regulation of fat cell differentiation	IL6,TGFB1,SIRT1,TNF	4	3.53E-05	59.13	0.00116
GO BP	oxidative demethylation	CYP2C8,CYP1A2,CYP3A4	3	3.95E-05	295.64	0.00121
GO BP	toxin metabolic process	CYP1A2,CYP1A1,CYP1B1	3	3.95E-05	295.64	0.00121
GO BP	cellular response to hydrogen peroxide	IL6,CYP1B1,SIRT1,NFE2L2	4	5.86E-05	49.97	0.00174
GO BP	regulation of cell population proliferation	TGFB1,STAT3,SIRT1,TNF,EGFR	5	6.04E-05	21.74	0.00174
GO BP	inflammatory response to wounding	IL6,STAT3,TNF	3	7.23E-05	221.73	0.00196
GO BP	negative regulation of lipid storage	IL6,PPARG,TNF	3	7.23E-05	221.73	0.00196
GO BP	negative regulation of cell population proliferation	IL6,TGFB1,STAT3,BCL2,CYP1B1,TNF	6	9.57E-05	11.83	0.00252
GO BP	response to estradiol	TGFB1,ABCB1,CASP3,STAT3	4	0.000107	40.78	0.00275
GO BP	positive regulation of DNA-templated transcription	IL6,TGFB1,STAT3,PPARG,PPARA,TNF,NFE2L2	7	0.00014	7.85	0.00349
GO BP	response to vitamin A	ABCB1,CYP1A1,PPARG	3	0.000149	156.51	0.00361
GO BP	negative regulation of collagen biosynthetic process	IL6,PPARG,CYP7A1	3	0.000167	147.82	0.00396
GO BP	positive regulation of interleukin-6 production	IL6,TGFB1,STAT3,TNF	4	0.000177	34.44	0.0041
GO BP	epithelial cell apoptotic process	CASP3,BCL2,HMOX1	3	0.000207	133.04	0.00468
GO BP	positive regulation of cell population proliferation	IL6,TGFB1,STAT3,BCL2,SIRT1,EGFR	6	0.000228	9.82	0.00496
GO BP	negative regulation of apoptotic process	IL6,MAPK8,BCL2,SIRT1,TNF,EGFR	6	0.00023	9.80	0.00496

GO BP	response to immobilization stress	TGFB1,CYP1A1,PPARG	3	0.0003	110.86	0.00632
GO BP	retinoic acid metabolic process	CYP2C8,UGT1A1,CYP3A4	3	0.000353	102.34	0.00728
GO BP	hormone metabolic process	CYP1A2,CYP1A1,CYP1B1	3	0.00041	95.03	0.00827
GO BP	liver regeneration	IL6,TGFB1,TNF	3	0.00044	91.75	0.00869
GO BP	negative regulation of gene expression	TGFB1,STAT3,PPARG,SIRT1,TNF	5	0.000484	12.63	0.00936
GO BP	rhythmic process	MAPK8,PPARG,PPARA,SIRT1	4	0.000545	23.49	0.0103
GO BP	response to lipopolysaccharide	ABCB1,CASP3,CYP1A1,TNF	4	0.000694	21.63	0.0129
GO BP	positive regulation of multicellular organismal process	IL6,TGFB1,STAT3	3	0.000758	70.02	0.0136
GO BP	positive regulation of cytokine production involved in inflammatory response	IL6,STAT3,TNF	3	0.000758	70.02	0.0136
GO BP	intracellular receptor signaling pathway	STAT3,PPARG,PPARA	3	0.000798	68.22	0.0138
GO BP	T cell homeostasis	TGFB1,CASP3,BCL2	3	0.000798	68.22	0.0138
GO BP	response to nicotine	CASP3,BCL2,HMOX1	3	0.000882	64.90	0.0149
GO BP	regulation of developmental process	SIRT1,TNF,EGFR	3	0.000926	63.35	0.0153
GO BP	arachidonate metabolic process	CYP2C8,CYP1A2,CYP1B1	3	0.00097	61.88	0.0153
GO BP	cellular response to ionizing radiation	TGFB1,SIRT1,TNF	3	0.00097	61.88	0.0153
GO BP	negative regulation of blood vessel endothelial cell migration	TGFB1,PPARG,TNF	3	0.00097	61.88	0.0153
GO BP	cellular response to lipopolysaccharide	IL6,MAPK8,ABCB1,TNF	4	0.00113	18.29	0.0175
GO BP	positive regulation of chemokine production	IL6,HMOX1,TNF	3	0.00126	54.30	0.0192
GO BP	positive regulation of blood vessel endothelial cell migration	TGFB1,SIRT1,NFE2L2	3	0.00131	53.21	0.0197
GO BP	cellular response to glucose starvation	BCL2,SIRT1,NFE2L2	3	0.00142	51.17	0.021
GO BP	inflammatory response	IL6,TGFB1,STAT3,TNF,NFE2L2	5	0.00148	9.36	0.0216
GO BP	positive regulation of phosphatidylinositol 3-kinase/protein kinase B signal transduction	TGFB1,SIRT1,TNF,EGFR	4	0.00156	16.35	0.0224
GO BP	intrinsic apoptotic signaling pathway in response to DNA damage	BCL2,SIRT1,TNF	3	0.00164	47.51	0.0232

GO BP	negative regulation of cytokine production involved in inflammatory response	STAT3,HMOX1,PPARA	3	0.0017	46.68	0.0234
GO BP	positive regulation of MAPK cascade	IL6,TGFB1,TNF,EGFR	4	0.0017	15.84	0.0234
GO BP	negative regulation of cell cycle	TGFB1,CASP3,SIRT1	3	0.00182	45.10	0.0246
GO BP	positive regulation of canonical NF-kappaB signal transduction	TGFB1,STAT3,HMOX1,TNF	4	0.00198	15.03	0.0264
GO BP	humoral immune response	IL6,BCL2,TNF	3	0.00207	42.23	0.0273
GO BP	energy homeostasis	MAPK8,STAT3,SIRT1	3	0.00214	41.57	0.0277
GO BP	response to ischemia	STAT3,BCL2,NFE2L2	3	0.0022	40.93	0.0282
GO BP	cell surface receptor signaling pathway via JAK-STAT	IL6,STAT3,TNF	3	0.00234	39.71	0.0292
GO BP	positive regulation of interleukin-8 production	IL6,STAT3,TNF	3	0.00234	39.71	0.0292
GO BP	response to mechanical stimulus	MAPK8,PPARG,TNF	3	0.00248	38.56	0.0297
GO BP	positive regulation of protein phosphorylation	SIRT1,TNF,EGFR	3	0.00248	38.56	0.0297
GO BP	regulation of circadian rhythm	MAPK8,PPARG,PPARA	3	0.00248	38.56	0.0297
GO BP	positive regulation of interleukin-1 beta production	IL6,STAT3,TNF	3	0.00255	38.01	0.0302
GO BP	cell population proliferation	TGFB1,STAT3,BCL2,EGFR	4	0.00281	13.29	0.0329
GO BP	heart development	TGFB1,CASP3,PPARG,PPARA	4	0.00321	12.67	0.0364
GO BP	dibenzo-p-dioxin metabolic process	CYP1A2,CYP1A1	2	0.00323	591.27	0.0364
GO BP	negative regulation of primary miRNA processing	IL6,STAT3	2	0.00323	591.27	0.0364
GO BP	gene expression	TGFB1,STAT3,PPARA,NFE2L2	4	0.00328	12.58	0.0365
GO BP	response to nutrient levels	ABCB1,PPARG,TNF	3	0.0034	32.85	0.0374
GO BP	female pregnancy	TGFB1,ABCB1,BCL2	3	0.00348	32.45	0.0379
GO BP	circadian rhythm	ABCB1,SIRT1,TNF	3	0.00365	31.68	0.0388
GO BP	response to toxic substance	BCL2,CYP1A1,CYP1B1	3	0.00365	31.68	0.0388
GO BP	negative regulation of transcription by RNA polymerase II	TGFB1,STAT3,PPARG,PPARA,SIRT1,TNF	6	0.00413	5.15	0.0436
GO BP	positive regulation of mononuclear cell migration	TGFB1,TNF	2	0.0043	443.45	0.0448

GO BP	phosphatidylinositol 3-kinase/protein kinase B signal transduction	STAT3,TNF,EGFR	3	0.00454	28.31	0.0468
KEGG	Lipid and atherosclerosis	IL6,CYP2C8,MAPK8,CASP3,STAT3,BCL2,CYP1A1,PPARG,TNF,NFE2L2	10	3.2E-10	19.98	3.87E-08
KEGG	Chemical carcinogenesis - receptor activation	UGT1A1,STAT3,CYP1A2,BCL2,CYP1A1,CYP1B1,CYP3A4,PPARA,EGFR	9	1.03E-08	17.90	6.23E-07
KEGG	AGE-RAGE signaling pathway in diabetic complications	IL6,MAPK8,TGFB1,CASP3,STAT3,BCL2,TNF	7	5.94E-08	29.92	2.4E-06
KEGG	Steroid hormone biosynthesis	UGT1A1,CYP1A2,CYP1A1,CYP1B1,CYP3A4,CYP7A1	6	2.22E-07	40.47	6.72E-06
KEGG	Chemical carcinogenesis - DNA adducts	CYP2C8,UGT1A1,CYP1A2,CYP1A1,CYP1B1,CYP3A4	6	3.76E-07	36.48	9.09E-06
KEGG	Non-alcoholic fatty liver disease	IL6,MAPK8,TGFB1,CASP3,PPARG,PPARA,TNF	7	8.2E-07	19.24	1.43E-05
KEGG	Pathways in cancer	IL6,MAPK8,TGFB1,CASP3,STAT3,BCL2,HMOX1,PPARG,EGFR,NFE2L2	10	8.28E-07	8.10	1.43E-05
KEGG	Hepatitis B	IL6,MAPK8,TGFB1,CASP3,STAT3,BCL2,TNF	7	1.02E-06	18.54	1.55E-05
KEGG	MicroRNAs in cancer	ABCB1,CASP3,STAT3,BCL2,CYP1B1,HMOX1,SIRT1,EGFR	8	3.58E-06	10.79	4.42E-05
KEGG	Toxoplasmosis	MAPK8,TGFB1,CASP3,STAT3,BCL2,TNF	6	3.65E-06	23.12	4.42E-05
KEGG	Chemical carcinogenesis - reactive oxygen species	MAPK8,CYP1A2,CYP1A1,CYP1B1,HMOX1,EGFR,NFE2L2	7	7.02E-06	13.31	7.72E-05
KEGG	FoxO signaling pathway	IL6,MAPK8,TGFB1,STAT3,SIRT1,EGFR	6	8.49E-06	19.47	8.57E-05
KEGG	Alcoholic liver disease	IL6,MAPK8,CASP3,PPARA,SIRT1,TNF	6	1.25E-05	17.98	0.000113
KEGG	Retinol metabolism	CYP2C8,UGT1A1,CYP1A2,CYP1A1,CYP3A4	5	1.31E-05	31.74	0.000113
KEGG	Metabolism of xenobiotics by cytochrome P450	UGT1A1,CYP1A2,CYP1A1,CYP1B1,CYP3A4	5	2.38E-05	27.32	0.000192
KEGG	Colorectal cancer	MAPK8,TGFB1,CASP3,BCL2,EGFR	5	3.49E-05	24.81	0.000264
KEGG	Tuberculosis	IL6,MAPK8,TGFB1,CASP3,BCL2,TNF	6	3.88E-05	14.23	0.000268
KEGG	Bile secretion	ABCB1,UGT1A1,ABCB11,CYP3A4,CYP7A1	5	3.99E-05	23.98	0.000268
KEGG	Epstein-Barr virus infection	IL6,MAPK8,CASP3,STAT3,BCL2,TNF	6	6.86E-05	12.63	0.000437
KEGG	Insulin resistance	IL6,MAPK8,STAT3,PPARA,TNF	5	8.46E-05	19.80	0.000505
KEGG	HIF-1 signaling pathway	IL6,STAT3,BCL2,HMOX1,EGFR	5	8.76E-05	19.62	0.000505

KEGG	Measles	IL6,MAPK8,CASP3,STAT3,BCL2	5	0.000217	15.53	0.00119
KEGG	Fluid shear stress and atherosclerosis	MAPK8,BCL2,HMOX1,TNF,NFE2L2	5	0.000235	15.20	0.00124
KEGG	Hepatitis C	CASP3,STAT3,PPARA,TNF,EGFR	5	0.000363	13.57	0.00183
KEGG	Inflammatory bowel disease	IL6,TGFB1,STAT3,TNF	4	0.00039	26.16	0.00189
KEGG	Adipocytokine signaling pathway	MAPK8,STAT3,PPARA,TNF	4	0.000464	24.66	0.00216
KEGG	Drug metabolism - cytochrome P450	CYP2C8,UGT1A1,CYP1A2,CYP3A4	4	0.000525	23.65	0.00235
KEGG	Pancreatic cancer	MAPK8,TGFB1,STAT3,EGFR	4	0.000614	22.42	0.00265
KEGG	Pertussis	IL6,MAPK8,CASP3,TNF	4	0.000637	22.14	0.00266
KEGG	EGFR tyrosine kinase inhibitor resistance	IL6,STAT3,BCL2,EGFR	4	0.000686	21.58	0.00277
KEGG	Proteoglycans in cancer	TGFB1,CASP3,STAT3,TNF,EGFR	5	0.000929	10.58	0.00363
KEGG	IL-17 signaling pathway	IL6,MAPK8,CASP3,TNF	4	0.00113	18.17	0.00428
KEGG	Endocrine resistance	MAPK8,BCL2,ABCB11,EGFR	4	0.00128	17.44	0.00468
KEGG	Human cytomegalovirus infection	IL6,CASP3,STAT3,TNF,EGFR	5	0.00138	9.51	0.00481
KEGG	Chagas disease	IL6,MAPK8,TGFB1,TNF	4	0.00143	16.76	0.00481
KEGG	Amoebiasis	IL6,TGFB1,CASP3,TNF	4	0.00143	16.76	0.00481
KEGG	Coronavirus disease - COVID-19	IL6,MAPK8,STAT3,TNF,EGFR	5	0.00165	9.07	0.00536
KEGG	Th17 cell differentiation	IL6,MAPK8,TGFB1,STAT3	4	0.00168	15.84	0.00536
KEGG	Linoleic acid metabolism	CYP2C8,CYP1A2,CYP3A4	3	0.00195	43.16	0.00605
KEGG	Salmonella infection	IL6,MAPK8,CASP3,BCL2,TNF	5	0.002	8.60	0.00605
KEGG	TNF signaling pathway	IL6,MAPK8,CASP3,TNF	4	0.00216	14.51	0.00639
KEGG	Apoptosis - multiple species	MAPK8,CASP3,BCL2	3	0.00222	40.47	0.0064
KEGG	Apoptosis	MAPK8,CASP3,BCL2,TNF	4	0.00323	12.60	0.00909
KEGG	Osteoclast differentiation	MAPK8,TGFB1,PPARG,TNF	4	0.00365	12.07	0.01
KEGG	Tryptophan metabolism	CYP1A2,CYP1A1,CYP1B1	3	0.0038	30.83	0.01
KEGG	MAPK signaling pathway	MAPK8,TGFB1,CASP3,TNF,EGFR	5	0.00382	7.19	0.01

KEGG	Gastric cancer	TGFB1,ABCB1,BCL2,EGFR	4	0.00417	11.51	0.0107
KEGG	Efferocytosis	TGFB1,CASP3,PPARG,SIRT1	4	0.00474	11.00	0.0119
KEGG	Necroptosis	MAPK8,STAT3,BCL2,TNF	4	0.00491	10.86	0.0121
KEGG	Malaria	IL6,TGFB1,TNF	3	0.00535	25.90	0.013
KEGG	JAK-STAT signaling pathway	IL6,STAT3,BCL2,EGFR	4	0.00572	10.28	0.0136
KEGG	Hepatocellular carcinoma	TGFB1,HMOX1,EGFR,NFE2L2	4	0.00591	10.16	0.0138
KEGG	Legionellosis	IL6,CASP3,TNF	3	0.00668	23.12	0.0152
KEGG	Herpes simplex virus 1 infection	IL6,CASP3,BCL2,TNF	4	0.00725	9.43	0.0162
KEGG	NOD-like receptor signaling pathway	IL6,MAPK8,BCL2,TNF	4	0.00793	9.14	0.0174
KEGG	Kaposi sarcoma-associated herpesvirus infection	IL6,MAPK8,CASP3,STAT3	4	0.00876	8.81	0.0189
KEGG	Pathogenic Escherichia coli infection	IL6,MAPK8,CASP3,TNF	4	0.00964	8.51	0.0205
KEGG	Epithelial cell signaling in Helicobacter pylori infection	MAPK8,CASP3,EGFR	3	0.0106	18.24	0.022
KEGG	Human immunodeficiency virus 1 infection	MAPK8,CASP3,BCL2,TNF	4	0.0111	8.07	0.0228
KEGG	PPAR signaling pathway	PPARG,PPARA,CYP7A1	3	0.012	17.04	0.0243
KEGG	Human T-cell leukemia virus 1 infection	IL6,MAPK8,TGFB1,TNF	4	0.0126	7.71	0.025
KEGG	Shigellosis	MAPK8,BCL2,TNF,EGFR	4	0.0174	6.82	0.034
KEGG	Rheumatoid arthritis	IL6,TGFB1,TNF	3	0.0184	13.63	0.0353
KEGG	Pathways of neurodegeneration - multiple diseases	IL6,MAPK8,CASP3,BCL2,TNF	5	0.0198	4.47	0.037
KEGG	Hypertrophic cardiomyopathy	IL6,TGFB1,TNF	3	0.0199	13.08	0.037
KEGG	C-type lectin receptor signaling pathway	IL6,MAPK8,TNF	3	0.0222	12.33	0.0404
KEGG	Prion disease	IL6,MAPK8,CASP3,TNF	4	0.0223	6.21	0.0404
KEGG	Toll-like receptor signaling pathway	IL6,MAPK8,TNF	3	0.0238	11.88	0.0423

Table S3. KEGG pathway enrichment analysis of the 22 core targets of VP

Category	Term	Genes	Count	P-Value	Fold Enrichment	FDR
GO BP	positive regulation of canonical NF-kappaB signal transduction	PRKN,PINK1,STAT3,HMOX1,TNF	5	1.41E-06	45.93	0.000547
GO BP	response to xenobiotic stimulus	PRKN,CASP3,STAT3,TNF,NFE2L2	5	2.07E-06	41.69	0.000547
GO BP	regulation of cellular response to oxidative stress	PRKN,PINK1,NFE2L2	3	8.08E-06	591.27	0.00103
GO BP	cellular response to toxic substance	PRKN,PINK1,TNF	3	9.69E-06	542.00	0.00103
GO BP	inflammatory response to wounding	IL6,STAT3,TNF	3	9.69E-06	542.00	0.00103
GO BP	positive regulation of ATP biosynthetic process	PRKN,PINK1,STAT3	3	1.54E-05	433.60	0.00136
GO BP	response to oxidative stress	PRKN,PINK1,HMOX1,NFE2L2	4	2.33E-05	58.59	0.00176
GO BP	positive regulation of gene expression	PRKN,IL6,STAT3,TNF,NFE2L2	5	3.09E-05	21.05	0.00204
GO BP	positive regulation of transcription by RNA polymerase II	PRKN,IL6,PINK1,STAT3,TNF,NFE2L2	6	4.96E-05	10.47	0.00272
GO BP	regulation of reactive oxygen species metabolic process	PRKN,PINK1,TNF	3	5.14E-05	240.89	0.00272
GO BP	positive regulation of cytokine production involved in inflammatory response	IL6,STAT3,TNF	3	0.000103	171.16	0.00494

GO BP	positive regulation of chemokine production	IL6,HMOX1,TNF	3	0.000171	132.73	0.00755
GO BP	positive regulation of smooth muscle cell proliferation	IL6,HMOX1,TNF	3	0.000193	125.08	0.00786
GO BP	response to glucocorticoid	IL6,CASP3,TNF	3	0.000208	120.44	0.00787
GO BP	positive regulation of apoptotic process	PRKN,IL6,CASP3,TNF	4	0.000259	26.04	0.00849
GO BP	positive regulation of miRNA transcription	IL6,STAT3,TNF	3	0.000266	106.62	0.00849
GO BP	response to ischemia	PINK1,STAT3,NFE2L2	3	0.000302	100.06	0.00849
GO BP	negative regulation of gene expression	PRKN,PINK1,STAT3,TNF	4	0.000302	24.71	0.00849
GO BP	cell surface receptor signaling pathway via JAK-STAT	IL6,STAT3,TNF	3	0.000321	97.07	0.00849
GO BP	positive regulation of interleukin-8 production	IL6,STAT3,TNF	3	0.000321	97.07	0.00849
GO BP	positive regulation of interleukin-1 beta production	IL6,STAT3,TNF	3	0.00035	92.91	0.00882
GO BP	macroautophagy	PRKN,PINK1,HMOX1	3	0.000435	83.38	0.0105
GO BP	inflammatory response	IL6,STAT3,TNF,NFE2L2	4	0.000728	18.30	0.0167
GO BP	positive regulation of interleukin-6 production	IL6,STAT3,TNF	3	0.000757	63.15	0.0167
GO BP	cellular response to oxidative stress	PRKN,PINK1,NFE2L2	3	0.00091	57.56	0.0193
GO BP	modulation of chemical synaptic transmission	PRKN,STAT3,TNF	3	0.00108	52.88	0.021
GO BP	protein ubiquitination	PRKN,PINK1,ABCB11,NFE2L2	4	0.00112	15.77	0.021
GO BP	cellular response to hydrogen sulfide	PRKN,PINK1	2	0.00123	1445.33	0.021
GO BP	negative regulation of primary miRNA processing	IL6,STAT3	2	0.00123	1445.33	0.021

GO BP	mitochondrion to lysosome vesicle-mediated transport	PRKN,PINK1	2	0.00123	1445.33	0.021
GO BP	regulation of synaptic vesicle transport	PRKN,PINK1	2	0.00123	1445.33	0.021
GO BP	positive regulation of angiogenesis	STAT3,HMOX1,NFE2L2	3	0.00181	40.65	0.0299
GO BP	negative regulation of mitochondrial fission	PRKN,PINK1	2	0.00205	867.20	0.0328
GO BP	response to hypoxia	CASP3,STAT3,TNF	3	0.00231	35.93	0.0359
GO BP	vascular endothelial growth factor production	IL6,TNF	2	0.00246	722.67	0.0371
GO BP	positive regulation of DNA-templated transcription	IL6,STAT3,TNF,NFE2L2	4	0.00319	10.96	0.0468
GO BP	regulation of protein targeting to mitochondrion	PRKN,PINK1	2	0.00328	542.00	0.0468
KEGG	Lipid and atherosclerosis	IL6,CASP3,STAT3,TNF,NFE2L2	5	0.000017	24.42	0.00141
KEGG	AGE-RAGE signaling pathway in diabetic complications	IL6,CASP3,STAT3,TNF	4	6.29E-05	41.79	0.00261
KEGG	Hepatitis B	IL6,CASP3,STAT3,TNF	4	0.000261	25.89	0.00722
KEGG	Pathways of neurodegeneration - multiple diseases	PRKN,IL6,PINK1,CASP3,TNF	5	0.000393	10.92	0.00793
KEGG	Epstein-Barr virus infection	IL6,CASP3,STAT3,TNF	4	0.000513	20.59	0.00793
KEGG	Pathways in cancer	IL6,CASP3,STAT3,HMOX1,NFE2L2	5	0.000573	9.90	0.00793
KEGG	Human cytomegalovirus infection	IL6,CASP3,STAT3,TNF	4	0.000691	18.59	0.00819
KEGG	Legionellosis	IL6,CASP3,TNF	3	0.000935	56.52	0.0097
KEGG	Parkinson disease	PRKN,PINK1,CASP3,NFE2L2	4	0.00116	15.57	0.0107

KEGG	Inflammatory bowel disease	IL6,STAT3,TNF	3	0.0013	47.96	0.0108
KEGG	Pertussis	IL6,CASP3,TNF	3	0.00181	40.58	0.0136
KEGG	IL-17 signaling pathway	IL6,CASP3,TNF	3	0.00267	33.32	0.018
KEGG	Amyotrophic lateral sclerosis	PRKN,PINK1,CASP3,TNF	4	0.00286	11.38	0.018
KEGG	Amoebiasis	IL6,CASP3,TNF	3	0.00313	30.73	0.018
KEGG	Insulin resistance	IL6,STAT3,TNF	3	0.00349	29.04	0.018
KEGG	HIF-1 signaling pathway	IL6,STAT3,HMOX1	3	0.00356	28.78	0.018
KEGG	Toxoplasmosis	CASP3,STAT3,TNF	3	0.00369	28.26	0.018
KEGG	TNF signaling pathway	IL6,CASP3,TNF	3	0.00415	26.60	0.0191
KEGG	Measles	IL6,CASP3,STAT3	3	0.00562	22.77	0.0238
KEGG	Fluid shear stress and atherosclerosis	HMOX1,TNF,NFE2L2	3	0.00586	22.29	0.0238
KEGG	Alcoholic liver disease	IL6,CASP3,TNF	3	0.00602	21.98	0.0238
KEGG	Non-alcoholic fatty liver disease	IL6,CASP3,TNF	3	0.00712	20.16	0.0263
KEGG	Hepatitis C	CASP3,STAT3,TNF	3	0.0073	19.91	0.0263
KEGG	Influenza A	IL6,CASP3,TNF	3	0.0086	18.30	0.0297
KEGG	Tuberculosis	IL6,CASP3,TNF	3	0.00948	17.39	0.0306
KEGG	Herpes simplex virus 1 infection	IL6,CASP3,TNF	3	0.00958	17.30	0.0306
KEGG	Kaposi sarcoma-associated herpesvirus infection	IL6,CASP3,STAT3	3	0.0109	16.15	0.0336

KEGG	Pathogenic Escherichia coli infection	IL6,CASP3,TNF	3	0.0117	15.59	0.0338
KEGG	Proteoglycans in cancer	CASP3,STAT3,TNF	3	0.0118	15.52	0.0338
KEGG	Coronavirus disease - COVID-19	IL6,STAT3,TNF	3	0.0159	13.30	0.0439
KEGG	Salmonella infection	IL6,CASP3,TNF	3	0.0175	12.61	0.047

Table S4. Herbal composition of hepatoprotective reference prescriptions used in the comparative analysis

Prescription	Herbal components (Latin pharmacognostic name)
Saenggan-geonbi-tang	*Artemisiae Capillaris Herba*; *Alismatis Rhizoma*; *Atractylodis Rhizoma Alba*; *Poria Cocos*; *Polyporus*; *Crataegi Fructus*; *Hordei Fructus Germinatus*; *Citri Reticulatae Pericarpium*; *Magnoliae Cortex*; *Aurantii Fructus Immaturus*; *Atractylodis Rhizoma*; *Agastachis Herba*; *Pinelliae Rhizoma*; *Arecae Pericarpium*; *Amomi Fructus*; *Zingiberis Rhizoma Recens*; *Gentianae Radix*; *Massa Medicata Fermentata*; *Sparganii Rhizoma*; *Curcumae Rhizoma*; *Raphani Semen*; *Glycyrrhizae Radix*
Injinoryeong-san	*Artemisiae Capillaris Herba*; *Alismatis Rhizoma*; *Poria Cocos*; *Polyporus*; *Atractylodis Rhizoma Alba*; *Cinnamomi Ramulus*
Hwangnyeonhaedok-tang	*Coptidis Rhizoma*; *Scutellariae Radix*; *Phellodendri Cortex*; *Gardeniae Fructus*
Yongdamsagan-tang	*Gentianae Radix*; *Bupleuri Radix*; *Alismatis Rhizoma*; *Plantaginis Semen*; *Akebiae Caulis*; *Scutellariae Radix*; *Gardeniae Fructus*; *Angelicae Gigantis Radix*; *Rehmanniae Radix*; *Glycyrrhizae Radix*

Table S5. Comprehensive list of 158 candidate compounds in VP and their database mapping status

No.	Herb	Compound	InChIKey	SMILES	Source	STITCH Mapping
**1**	Phellodendri Cortex	berberine	OJDYKDYYXBXBIX-UHFFFAOYSA-C2:C149N	C1C[N+]2=C(C=C3C=CC4=C(C3=C2)OCO4)C5=CC6=C(C=C51)OCO6	TCMSP	Excluded
**2**	Phellodendri Cortex Scutellariae Radix	coptisine	RMOPHAINWJSBTC-UHFFFAOYSA-N	CC(=CCOC1=C2C(=C(C3=C1OC(=O)C=C3)OC)C=CO2)C	TCMSP	Excluded
**3**	Phellodendri Cortex	(±)-lyoniresinol	ZDVZKBOFCHOPLM-QWQRMKEZSA-N	COC1=CC(=CC(=C1O)OC)[C@@H]2[C@H]([C@H](CC3=CC(=C(C(=C23)OC)O)OC)CO)CO	TCMSP	Included
**4**	Phellodendri Cortex	Obacunoic acid	GUIYORVNNIUEBH-LJHARGAWSA-N	C[C@@]12CC[C@@H]3[C@@]([C@@H](CC(=O)[C@]3([C@@]14[C@H](O4)C(=O)O[C@H]2C5=COC=C5)C)C(C)(C)O)(C)/C=C\C(=O)O	TCMSP	Included
**5**	Phellodendri Cortex	Obacunone	MAYJEFRPIKEYBL-OASIGRBWSA-N	C[C@@]12CC[C@@H]3[C@]4(C=CC(=O)OC([C@@H]4CC(=O)[C@]3([C@@]15[C@H](O5)C(=O)O[C@H]2C6=COC=C6)C)(C)C)C	TCMSP	Included
**6**	Phellodendri Cortex	Phellavin_qt	MSWZSCFWXOMBLH-RBUKOAKNSA-N	N/A	TCMSP	Excluded
**7**	Phellodendri Cortex	phellodendrine	KEPMLIRTYOVGET-INIZCTEOSA-N	COC1=C(C=CC(=C1)CO)O[C@H]2[C@@H]([C@H]([C@@H]([C@H](O2)CO)O)O)O	TCMSP	Excluded
**8**	Phellodendri Cortex	Phellopterin	BMLZFLQMBMYVHG-UHFFFAOYSA-N	C[C@@]12CC[C@@H]3[C@@]([C@@H](CC(=O)[C@]3([C@@]14[C@H](O4)C(=O)O[C@H]2C5=COC=C5)C)C(C)(C)O)(C)/C=C\C(=O)O	TCMSP	Included

**9**	Phellodendri Cortex	Vanilloloside	SIMPNXWTAVEOTO-MQLXINIDSA-N	C[C@@]12CC[C@@H]3[C@]4(C=CC(=O)OC([C@@H]4CC(=O)[C@]3([C@@]15[C@H](O5)C(=O)O[C@H]2C6=COC=C6)C)(C)C)C	TCMSP	Included
**10**	Phellodendri Cortex	Coniferin	SFLMUHDGSQZDOW-FAOXUISGSA-N	C[N@@+]12CCC3=CC(=C(C=C3[C@@H]1CC4=CC(=C(C=C4C2)OC)O)O)OC	TCMSP	Included
**11**	Phellodendri Cortex	Dehydrotanshinone II A	PURTYNPVRFEUEN-UHFFFAOYSA-N	C1C[C@@H]2CN3C(=O)C=CC=C3C4=CCCN([C@H]24)C1	TCMSP	Included
**12**	Phellodendri Cortex	delta7-Dehydrosophoramine	POPJONPBVXYYED-IAQYHMDHSA-N	CC(C)(CCC1=C2C(=C(C=C1O)O)C(=O)C(=C(O2)C3=CC=C(C=C3)O)O)O	TCMSP	Included
**13**	Phellodendri Cortex	Amurensin_qt	CTGVBHDTGZUEJZ-UHFFFAOYSA-N	CC1=COC2=C1C(=O)C(=O)C3=C2C=CC4=C3C=CCC4(C)C	TCMSP	Included
**14**	Phellodendri Cortex	hispidol B	MVWLZBQPRMCRKT-LUNRJGNMSA-N	N/A	TCMSP	Included
**15**	Phellodendri Cortex	kihadanin A	DZWMSKKYNYZQTC-BAVACYCRSA-N	C1CN2C(=NC3=CC=CC=C3C2=O)C4=C1C5=CC=CC=C5N4	TCMSP	Included
**16**	Phellodendri Cortex	rutaecarpine	ACVGWSKVRYFWRP-UHFFFAOYSA-N	CC1=C2C3=CC4=C(C=C3CC[N+]2=CC5=CC6=C(C=C15)OCO6)OCO4	TCMSP	Included
**17**	Phellodendri Cortex	Skimmianin	SLSIBLKBHNKZTB-UHFFFAOYSA-N	COC1=C(C2=C(C=C1)C(=C3C=COC3=N2)OC)OC	TCMSP	Included
**18**	Phellodendri Cortex	Chelerythrine	FYNOOIHIAAKWCH-UHFFFAOYSA-N	C[C@@H]1[C@@H]2C3=CC(=C(C=C3CCN2CC4=C1C=CC5=C4OCO5)OC)OC	TCMSP	Excluded
**19**	Phellodendri Cortex	Worenine	NYVARXJCEOLWKL-UHFFFAOYSA-N	C[C@@]12CC[C@@H]3[C@]4(C=CC(=O)OC([C@@H]4CC(=O)[C@]3([C@@]15[C@H](O5)C(=O)O[C@H]2C6=CC(=O)OC6O)C)(C)C)C	TCMSP	Excluded
**20**	Phellodendri Cortex	Cavidine	JTZZGWPIBBTYNE-FKIZINRSSA-N	C[N+]1=C2C(=C3C=CC(=C(C3=C1)OC)OC)C=CC4=CC5=C(C=C42)OCO5	TCMSP	Included
**21**	Phellodendri Cortex	Hispidone	YIBXWXOYFGZLRU-IDBNLLBVSA-N	C1=CC(=CC=C1[C@H]2[C@@H](C(=O)C3=C(C=C(C=C3O2)O)O)O)O	TCMSP	Included

**22**	Phellodendri Cortex	Syrigin	QJVXKWHHAMZTBY-GCPOEHJPSA-N	C1=CC(=C(C(C=C1C2=C(C(=O)C3=C(C=C(C=C3O2)O)O)O)O)O)O	TCMSP	Included
**23**	Phellodendri Cortex	Magnograndiolide	VHFXPBHLQOPQHJ-ABBQYLIMSA-N	C1=CC(=C(C(C=C1C2=C(C(=O)C3=C(C=C(C=C3O2)O)O)O[C@H]4[C@@H]([C@H]([C@H]([C@H](O4)CO)O)O)O)O)O	TCMSP	Included
**24**	Phellodendri Cortex	(2S,3S)-3,5,7-trihydroxy-2-(4-hydroxyphenyl)chroman-4-one	PADQINQHPQKXNL-CABCVRRESA-N	COC1=C(C2=C(C[C@@H]3C4=CC(=C(C=C4CCN3C2)OC)OC)C=C1)OC	TCMSP	Included
**25**	Phellodendri Cortex	Menisporphine	LHRMNRPHVHDOJH-UHFFFAOYSA-N	CN1CCC2=CC3=C(C=C2C(=O)CC4=C(C1)C5=C(C=C4)OCO5)OCO3	TCMSP	Included
**26**	Phellodendri Cortex	palmatine	DJDOMQTVQYDGJL-UHFFFAOYSA-N	COC1=C(C2=C(C[C@H]3C4=CC(=C(C=C4CCN3C2)OC)O)C=C1)OC	TCMSP	Excluded
**27**	Phellodendri Cortex	Fumarine	GPTFURBXHJWNHR-UHFFFAOYSA-N	COC1=C(C2=C[N+]3=C(C=C2C=C1)C4=CC(=C(C=C4CC3)OC)OC)OC	TCMSP	Included
**28**	Phellodendri Cortex	Isocorypalmine	KDFKJOFJHSVROC-INIZCTEOSA-N	COC1=CC2=C(C=C1)C3=NC=CC4=CC(=C(C(=C43)C2=O)OC)OC	TCMSP	Included
**29**	Phellodendri Cortex Sophorae Tonkinensis Radix	quercetin	REFJWTPEDVJJIY-UHFFFAOYSA-N	C[N+]1(CCC2=CC(=C(C3=C2[C@@H]1CC4=C3C(=C(C=C4)OC)O)O)OC)C	TCMSP	Included
**30**	Phellodendri Cortex	STOCK1N-14407	AEQDJSLRWYMAQI-QGZVFWFLSA-N	C[C@@]12CC[C@H]3C(=CC[C@H]4[C@@]3(CCC(=O)C4(C)C)C)[C@]1(CC[C@H]2[C@@H]5C[C@H]([C@@H](C(OC5)(C)C)O)O)C	TCMSP	Included
**31**	Phellodendri Cortex Scutellariae Radix	jatrorrizine	XXGZQSYRHXUIMO-UHFFFAOYSA-N	N/A	TCMSP	Excluded
**32**	Phellodendri Cortex	menisperine	LEYPLRBUVJSAAI-AWEZNQCLSA-N	C1=CC(=C(C(C=C1C2=C(C(=O)C3=C(C=C(C=C3O2)O)O)O[C@H]4[C@@H]([C@H]([C@H]([C@H](O4)CO)O)O)O)O)O	TCMSP	Excluded

**33**	Phellodendri Cortex	phellamurin_qt	IPWPEUJWMOPJDG-RBUKOAKNSA-N	CC(=CCC1=C2C(=C(C=C1O)O)C(=O)[C@@H]([C@H](O2)C3=CC=C(C=C3)O)O)C	TCMSP	Included
**34**	Phellodendri Cortex	(S)-Canadine	VZTUIEROBZXUFA-INIZCTEOSA-N	COC1=C(C2=C(C[C@H]3C4=CC5=C(C=C4CCN3C2)OCO5)C=C1)OC	TCMSP	Included
**35**	Phellodendri Cortex	columbamine	DIAORPOAESVSJD-UHFFFAOYSA-N	COC1=C(C2=C[N+]3=C(C=C2C=C1)C4=CC(=C(C=C4CC3)OC)O)OC	TCMSP	Excluded
**36**	Phellodendri Cortex	magnoflorine	YHJFRXGWUVSYOV-ZDUSSCGKSA-N	COC1=C(C2=C[N+]3=C(C=C2C=C1)C4=CC5=C(C=C4CC3)OCO5)O	TCMSP	Excluded
**37**	Phellodendri Cortex	berberrubine	VRYRNVUINYKGJK-UHFFFAOYSA-N	C[C@@]12CC[C@H]3[C@]([C@@]14[C@H](O4)C(=O)O[C@H]2C5=COC=C5)(C(=O)C[C@@H]6[C@@]37COC(=O)C[C@@H]7OC6(C)C)C	TCMSP	Excluded
**38**	Phellodendri Cortex	limonin	KBDSLGBFQAGHBE-MSGMIQHVSA-N	C[N+]1(CCC2=CC(=C(C3=C2[C@@H]1CC4=C3C(=C(C=C4)OC)O)O)OC)C	TCMSP	Included
**39**	Phellodendri Cortex	Hyperin	OVSQVDMCBVZWGM-DTGCRPNFSA-N	C[N@@+]12CCC3=CC(=C(C=C3[C@@H]1CC4=CC(=C(C=C4C2)OC)O)O)OC	TCMSP	Included
**40**	Phellodendri Cortex	SMR000232320	DCGUKHULKAAOPB-QBLSGNHRSA-N	C1=CC=C2C(=C1)C3=C4N2C(=O)C=CC4=NC=C3	TCMSP	Included
**41**	Phellodendri Cortex	Canthin-6-one	ZERVJPYNQLONEK-UHFFFAOYSA-N	CC1(C2CC=C3C(C2(CCC1=O)C)CCC4(C3(CCC4C5CC(OC5O)C6C(O6)(C)C)C)C)C	TCMSP	Included
**42**	Phellodendri Cortex	4-[(1R,3aS,4R,6aS)-4-(4-hydroxy-3,5-dimethoxyphenyl)-1,3,3a,4,6,6a-	KOWMJRJXZMEZLD-WRMVBYCNSA-N	COC1=CC(=CC(=C1O)OC)[C@H]2[C@@H]3CO[C@H]([C@@H]3CO2)C4=CC(=C(C(=C4)OC)O)OC	TCMSP	Included

		hexahydrofuro[4,3-c]furan-1-yl]-2,6-dimethoxyphenol				
**43**	Phellodendri Cortex	melianone	ZWXPNDUTGNVQEU-STEPOWRLSA-N	COC1=C(C=C(C2=C1OC(=CC2=O)C3=CC=CC=C3)O)O	TCMSP	Included
**44**	Phellodendri Cortex	thalifendine	SIWRWGMNOMKIKV-UHFFFAOYSA-N	COC1=C(C=C(C2=C1OC(=CC2=O)C3=CC=CC=C3)O)O[C@H]4[C@@H]([C@H]([C@@H]([C@H](O4)C(=O)O)O)O)O	TCMSP	Excluded
**45**	Phellodendri Cortex	vanilloloside	SIMPNXWTAVEOTO-RKQHYHRCSA-N	COC1=C(C=CC(=C1)CO)O[C@H]2[C@@H]([C@H]([C@@H]([C@H](O2)CO)O)O)O	TCMSP	Included
**46**	Scutellariae Radix	acacetin	DANYIYRPLHHOCZ-UHFFFAOYSA-N	COC1=C(C=CC2=CC3=[N+](CCC4=CC5=C(C=C43)OCO5)C=C21)O	TCMSP	Included
**47**	Scutellariae Radix	wogonin	XLTFNNCXVBYBSX-UHFFFAOYSA-N	COC1=CC=C(C=C1)C2=CC(=O)C3=C(C=C(C=C3O2)O)O	TCMSP	Included
**48**	Scutellariae Radix	Oroxindin	LNOHXHDWGCMVCO-NTKSAMNMSA-N	C[C@@H](C[C@H]([C@@H](C(C)(C)O)O)O)[C@@H]1CC[C@]2([C@]1(CC[C@H]3C2=CC[C@@H]4[C@@]3(CCC(=O)C4(C)C)C)C)C	TCMSP	Included
**49**	Scutellariae Radix	(2R)-7-hydroxy-5-methoxy-2-phenylchroman-4-one	QQQCWVDPMPFUGF-CYBMUJFWSA-N	COC1=CC(=CC2=C1C(=O)C[C@@H](O2)C3=CC=CC=C3)O	TCMSP	Included
**50**	Scutellariae Radix	chrysin	RTIXKCRFFJGDFG-UHFFFAOYSA-N	C1=CC(=CC=C1C2=CC(=O)C3=C(O2)C=C(C(=C3O)O)O)O	TCMSP	Included
**51**	Scutellariae Radix	baicalein	FXNFHKRTJBSTCS-UHFFFAOYSA-N	C1=CC=C(C=C1)C2=CC(=O)C3=C(C=C(C=C3O2)O)O	TCMSP	Included
**52**	Scutellariae Radix	scutellarein	JVXZRQGOGOXCEC-UHFFFAOYSA-N	C1=CC=C(C=C1)C2=CC(=O)C3=C(O2)C=C(C(=C3O)O)O	TCMSP	Included
**53**	Scutellariae Radix	5,8,2'-Trihydroxy-7-methoxyflavone	NKSITEVSJGHWCO-UHFFFAOYSA-N	COC1=C(C2=C(C(=C1)O)C(=O)C=C(O2)C3=CC=CC=C3O)O	TCMSP	Included
**54**	Scutellariae Radix	5,7,2,5-tetrahydroxy-8,6-dimethoxyflavone	XGJUUNRZFPFMOK-UHFFFAOYSA-N	COC1=C(C=CC(=C1C2=CC(=O)C3=C(C(=C(C(=C3O2)OC)O)OC)O)O)O	TCMSP	Included
**55**	Scutellariae Radix	Carthamidin	NPLTVGMLNDMOQE-LLVKDONJSA-N	C1[C@H](OC2=C(C1=O)C(=C(C(=C2)O)O)O)C3=CC=C(C=C3)O	TCMSP	Included

**56**	Scutellariae Radix	2,6,2',4'-tetrahydroxy-6'-methoxychaleone	XFUBPGKNMBCAHX-WAYWQWQTSA-N	C1[C@H](OC2=C(C1=O)C(=C(C(=C2)O)O)O)C3=CC=CC=C3	TCMSP	Included
**57**	Scutellariae Radix	Dihydrobaicalin	UVNUGBQJLDGZKE-ZKYDHBDKSA-N	C1[C@H](OC2=CC(=C(C(=C2C1=O)O)O)O[C@H]3[C@@H]([C@H]([C@@H]([C@H](O3)C(=O)O)O)O)O)C4=CC=CC=C4	TCMSP	Included
**58**	Scutellariae Radix	Dihydrobaicalin_qt	GPDJGLOROGNHJD-NSHDSACASA-N	C1[C@H](OC2=C(C1=O)C(=C(C(=C2)O)O)O)C3=CC=CC=C3	TCMSP	Included
**59**	Scutellariae Radix	Eriodyctiol (flavanone)	SBHXYTNGIZCORC-CYBMUJFWSA-N	C1=CC(=C(C(=C1)O)C2=C(C(=O)C3=C(C=C(C=C3O2)O)O)O)O	TCMSP	Included
**60**	Scutellariae Radix	Salvigenin	QCDYOIZVELGOLZ-UHFFFAOYSA-N	CCO[C@H]1[C@]([C@H]([C@@H]([C@H](O1)CO)O)O[C@H]2[C@@H]([C@@H]([C@H]([C@@H](O2)C)O)O)O)(C3=CC(=C(C=C3)OC)O)O	TCMSP	Included
**61**	Scutellariae Radix	2-(2,6-dihydroxyphenyl)-3,5,7-trihydroxy-chromone	NULZZCUABWZIRV-UHFFFAOYSA-N	C1[C@@H](OC2=CC(=CC(=C2C1=O)O)O)C3=CC(=C(C=C3)O)O	TCMSP	Included
**62**	Scutellariae Radix	5,2',6'-Trihydroxy-7,8-dimethoxyflavone	GWRFNPJGKCUUSJ-UHFFFAOYSA-N	COC1=C(C(=C(C=C1)C2=C(C(=O)C3=C(C=C(C=C3O2)O)O)O)OC)O	TCMSP	Included
**63**	Scutellariae Radix	Ganhuangenin	CPFJPTACQDZPLS-UHFFFAOYSA-N	COC1=C(C=CC(=C1C2=CC(=O)C3=C(O2)C(=C(C=C3O)O)OC)O)O	TCMSP	Included
**64**	Scutellariae Radix	Viscidulin III	LYWHJXVFIUKXLE-UHFFFAOYSA-N	COC1=C(C2=C(C(=C1)O)C(=O)C=C(O2)C3=C(C=CC=C3O)O)OC	TCMSP	Included
**65**	Scutellariae Radix	(2S,3R,4R,5R,6S)-2-[(2R,3R,4S,5R,6R)-3,5-dihydroxy-2-[2-(3-hydroxy-4-methoxy-phenyl)ethoxy]-6-methylol-tetrahydropyran-4-yl]oxy-6-methyl-tetrahydropyran-3,4,5-triol	MMRYSDPTALIPSP-MGXJDOAZSA-N	C[C@H]1[C@@H]([C@H]([C@H]([C@@H](O1)O[C@H]2[C@@H]([C@H](O[C@H]([C@@H]2O)OCCC3=CC(=C(C=C3)OC)O)CO)O)O)O)O	TCMSP	Included

**66**	Scutellariae Radix	darendoside B	ZLPXZRZUFKQINJ-GUEKMEBISA-N	COC1=CC=C(C=C1)C2=CC(=O)C3=C(C(=C(C=C3O2)OC)OC)O	TCMSP	Included
**67**	Scutellariae Radix	darendoside B_qt	XWBJWURSGCGEQR-MRLBHPIUSA-N	N/A	TCMSP	Excluded
**68**	Scutellariae Radix	5,7,2',6'-Tetrahydroxyflavone	WJXXUIYFPVIHDH-UHFFFAOYSA-N	C1=CC(=C(C(=C1)O)C2=CC(=O)C3=C(C=C(C=C3O2)O)O)O	TCMSP	Included
**69**	Scutellariae Radix	dihydrooroxylin A	GSTISHLARMKZOD-LBPRGKRZSA-N	C1=CC(=CC=C1C2=CC(=O)C3=C(C(=C(C=C3O2)O[C@H]4[C@@H]([C@H]([C@@H]([C@H](O4)C(=O)O)O)O)O)O)O)O	TCMSP	Excluded
**70**	Scutellariae Radix	Skullcapflavone II	GMQFOKBGMKVUQZ-UHFFFAOYSA-N	C1=CC(=CC=C1CCO[C@H]2[C@@H]([C@H]([C@@H]([C@H](O2)CO)O)O)O)O	TCMSP	Included
**71**	Scutellariae Radix	oroxylin a	LKOJGSWUMISDOF-UHFFFAOYSA-N	COC1=C(C2=C(C(=C1)O)C(=O)C=C(O2)C3=CC=CC=C3O)OC	TCMSP	Included
**72**	Scutellariae Radix	salidroside	ILRCGYURZSFMEG-MQLXINIDSA-N	COC1=C(C2=C(C=C1O)OC(=CC2=O)C3=CC=CC=C3)O	TCMSP	Included
**73**	Scutellariae Radix	scutellarin	DJSISFGPUUYILV-ZFORQUDYSA-N	COC1=C(C2=C(C=C1O)O[C@@H](CC2=O)C3=CC=CC=C3)O	TCMSP	Included
**74**	Scutellariae Radix	Panicolin	CZRGNFVQUYWGKP-UHFFFAOYSA-N	COC1=CC=CC(=C1C2=CC(=O)C3=C(C(=C(C(=C3O2)OC)OC)OC)O)O	TCMSP	Included
**75**	Scutellariae Radix	5,7,4'-Trihydroxy-8-methoxyflavone	OEZZJTAJYYSQKM-UHFFFAOYSA-N	COC1=C(C(=C2C(=C1O)C(=O)C=C(O2)C3=CC=CC=C3)O)OC	TCMSP	Included
**76**	Scutellariae Radix	NEOBAICALEIN	MIWLOORHWQBIHP-UHFFFAOYSA-N	C1=CC(=CC=C1C2=CC(=O)C3=C(C=C(C=C3O2)O)O)O	TCMSP	Excluded
**77**	Scutellariae Radix	Baicalin	DCWXTOWSMXDTNR-SPNOPHAYSA-N	C1=CC(=CC=C1C2=CC(=O)C3=C(C=C(C=C3O2)O[C@H]4[C@@H]([C@H]([C@@H]([C@H](O4)CO)O)O)O)O)O	TCMSP	Included
**78**	Scutellariae Radix	5,8-Dihydroxy-6,7-dimethoxyflavone	RXSYNUMMIWXLLC-UHFFFAOYSA-N	COC1=C(C(=C2C(=C1O)C(=O)C=C(O2)C3=CC=CC=C3O)OC)OC	TCMSP	Included
**79**	Scutellariae Radix	DIHYDROOROXYLIN	QUAPPCXFYKSDSV-GFCCVEGCSA-N	COC1=C(C2=C(C=C1O)O[C@H](CC2=O)C3=CC=CC=C3)O	TCMSP	Included
**80**	Scutellariae Radix	(+)-Syringaresinol	KOWMJRJXZMEZLD-HCIHMXRSSA-N	COC1=C(C=C(C2=C1OC(=CC2=O)C3=CC=C(C=C3)O)O)O	TCMSP	Included
**81**	Scutellariae Radix	Norwogonin	ZFKKRRMUPBBYRS-UHFFFAOYSA-N	C1=CC=C(C=C1)C2=CC(=O)C3=C(C(=C(C=C3O2)O[C@H]4[C@@H]([C@H]([C@@H]([C@H](O4)C(=O)O)O)O)O)O)O	TCMSP	Included

**82**	Scutellariae Radix	5,2'-Dihydroxy-6,7,8-trimethoxyflavone	QCKBVAGWPBRRQJ-UHFFFAOYSA-N	COC1=CC(=CC(=C1O)OC)[C@@H]2[C@H]3CO[C@@H]([C@H]3CO2)C4=CC(=C(C(=C4)OC)O)OC	TCMSP	Included
**83**	Scutellariae Radix	Cosmetin	KMOUJOKENFFTPU-QNDFHXLGSA-N	C1=CC=C(C=C1)C2=CC(=O)C3=C(O2)C(=C(C=C3O)O)O	TCMSP	Included
**84**	Scutellariae Radix	apigenin	KZNIFHPLKGYRTM-UHFFFAOYSA-N	C1=CO[C@H]([C@H]2[C@@H]1[C@@H]([C@H]3[C@@]2(O3)CO)O)O[C@H]4[C@@H]([C@H]([C@@H]([C@H](O4)CO)O)O)O	TCMSP	Included
**85**	Scutellariae Radix	ent-Epicatechin	PFTAWBLQPZVEMU-ZFWWWQNUSA-N	C1C[N+]2=C(C=C3C=CC4=C(C3=C2)OCO4)C5=CC6=C(C=C51)OCO6	TCMSP	Included
**86**	Scutellariae Radix	catalpol	XXGZQSYRHXUIMO-UHFFFAOYSA-N	C1[C@H]([C@@H]([C@@H](C[C@]1(C(=O)O)O)OC(=O)/C=C/C2=CC(=C(C=C2)O)O)O)O	TCMSP	Excluded
**87**	Scutellariae Radix	epiberberine	XXMJRBRPNZVNJR-UHFFFAOYSA-N	COC1=C(C2=C(C(=C1)O)C(=O)C=C(O2)C3=CC=CC=C3)OC	TCMSP	Excluded
**88**	Scutellariae Radix	5-o-caffeoylquinic acid	CWVRJTMFETXNAD-NXLLHMKUSA-N	COC1=C(C=C2C(=C1)CC[N+]3=C2C=C4C=CC5=C(C4=C3)OCO5)OC	TCMSP	Included
**89**	Scutellariae Radix	Moslosooflavone	IHLSBQVBFDTNTC-UHFFFAOYSA-N	COC1=CC=CC(=C1C2=CC(=O)C3=C(C(=C(C(=C3O2)OC)OC)OC)O)O	TCMSP	Included
**90**	Scutellariae Radix	2',3',5,7-tetrahydroxyflavone	STAGATUVRDVEAT-UHFFFAOYSA-N	C1=CC(=C(C(=C1)O)O)C2=CC(=O)C3=C(C=C(C=C3O2)O)O	TCMSP	Included
**91**	Scutellariae Radix	5,7,4'-trihydroxy-6-methoxyflavanone	FCXRFTLSXMRXTM-GFCCVEGCSA-N	COC1=C(C2=C(C=C1O)O[C@H](CC2=O)C3=CC=C(C=C3)O)O	TCMSP	Included
**92**	Scutellariae Radix	5,7,4'-trihydroxy-8-methoxyflavanone	OJCCBPWPNVUJFG-CYBMUJFWSA-N	N/A	TCMSP	Included
**93**	Scutellariae Radix	rivularin	IYMNRCWUEDWTPE-UHFFFAOYSA-N	COC1=CC=CC(=C1C2=CC(=O)C3=C(O2)C(=C(C=C3O)OC)OC)O	TCMSP	Included
**94**	Scutellariae Radix	Scutevulin	XCBMYKIKEHGYAR-UHFFFAOYSA-N	C1=CC(=C(C=C1C2=[O+]C3=CC(=CC(=C3C=C2O)O)O)O)O	TCMSP	Included
**95**	Aucklandiae Radix Phellodendri Cortex	Magnoflorine	TVTUVFVMZQZSMK-ZXZGSGMFSA-N	C1=CC(=C(C=C1CCO[C@H]2[C@@H]([C@H]([C@@H]([C@H](O2)COC(=O)/C=C/C3=CC(=C(C=C3)O)O)O)O)O)O)O	TCMSP	Excluded

**96**	Aucklandiae Radix	Calceolarioside B	LFKQVVDFNHDYNK-FOXCETOMSA-N	COC1=C(C=C(C2=C1OC(=CC2=O)C3=CC=CC=C3O)O)O	TCMSP	Included
**97**	Aucklandiae Radix	cyanidol	GFZJRQMBETXFQO-UHFFFAOYSA-N	COC1=CC=CC2=C3C(=C(C=C21)[N+](=O)[O-])C(=CC4=C3OCO4)C(=O)O	TCMSP	Excluded
**98**	Aucklandiae Radix	aristolochic acid A	KTEBZKXXNMHJFH-UHFFFAOYSA-N	C[N+]1(CCC2=CC(=C(C3=C2[C@@H]1CC4=C3C(=C(C=C4)OC)O)O)OC)C	TCMSP	Included
**99**	Aucklandiae Radix	Aristoloside_qt	QWSBJPJXYNNOKV-UHFFFAOYSA-N	N/A	TCMSP	Excluded
**100**	Aucklandiae Radix	aristolochic acidII	GUCQOMKSIUORSV-UHFFFAOYSA-N	N/A	TCMSP	Excluded
**101**	Aucklandiae Radix	Ariskanin A	PJSPBDYTEPZDNN-UHFFFAOYSA-N	N/A	TCMSP	Excluded
**102**	Aucklandiae Radix	quinatic acid	FKUBIEWSGBVADJ-GFFKOCLZSA-N	C[C@H](CCC(=O)NCC(=O)O)[C@H]1CC[C@@H]2[C@@]1([C@H](C[C@H]3[C@H]2CC[C@H]4[C@@]3(CC[C@H](C4)O)C)O)C	TCMSP	Included
**103**	Bovis Calculus	N-(3alpha,12alpha-dihydroxy-5beta-cholan-24-oyl)glycine	WVULKSPCQVQLCU-BUXLTGKBSA-N	C[C@H](CCC(=O)NCCS(=O)(=O)O)[C@H]1CC[C@@H]2[C@@]1([C@H](C[C@H]3[C@H]2CC[C@H]4[C@@]3(CC[C@H](C4)O)C)O)C	TCMSP	Included
**104**	Bovis Calculus	methyl (4R)-4-[(3R,5S,7S,8R,9S,10S,12S,13R,14S,17R)-3,7,12-trihydroxy-10,13-dimethyl-2,3,4,5,6,7,8,9,11,12,14,15,16,17-tetradecahydro-1H-cyclopenta[a]phenanthren-17-yl]pentanoate	DLYVTEULDNMQAR-GVFWELEYSA-N	C[C@H](CCC(=O)OC)[C@H]1CC[C@@H]2[C@@]1(CC[C@H]3[C@H]2[C@@H](C[C@H]4[C@@]3(CC[C@H](C4)O)C)O)C	TCMSP	Included
**105**	Bovis Calculus	Methyl desoxycholate	ZHUOOEGSSFNTNP-DMMQTPOTSA-N	C[C@H](CCC(=O)O)[C@H]1CC[C@@H]2[C@@]1(CC[C@H]3[C@H]2[C@@H](C[C@H]4[C@@]3(CC[C@H](C4)O)C)O)C	TCMSP	Included

**106**	Bovis Calculus	2-[(3alpha,12alpha-dihydroxy-24-oxo-5beta-cholan-24-yl)amino]ethanesulfonic acid	AWDRATDZQPNJFN-VAYUFCLWSA-N	C[C@H](CCC(=O)OC)[C@H]1CC[C@@H]2[C@@]1([C@H](C[C@H]3[C@H]2[C@H](C[C@H]4[C@@]3(CC[C@H](C4)O)C)O)O)C	TCMSP	Included
**107**	Bovis Calculus	chenodeoxycholic acid	RUDATBOHQWOJDD-BSWAIDMHSA-N	C[C@H](CCC(=O)O)[C@H]1CC[C@@H]2[C@@]1(CC[C@H]3[C@H]2[C@@H](C[C@H]4[C@@]3(CC[C@H](C4)O)C)O)C	TCMSP	Included
**108**	Bovis Calculus	methyl (4R)-4-[(3R,5S,7R,8R,9S,10S,13R,14S,17R)-3,7-dihydroxy-10,13-dimethyl-2,3,4,5,6,7,8,9,11,12,14,15,16,17-tetradecahydro-1H-cyclopenta[a]phenanthren-17-yl]pentanoate	GRQROVWZGGDYSW-IFJDUOSNSA-N	C[C@]12CC[C@H](C[C@H]1CC[C@@H]3[C@H]2[C@H](C[C@]4([C@@H]3CC[C@@H]4O)C)O)O	TCMSP	Included
**109**	Bovis Calculus	Deoxycholic Acid	KXGVEGMKQFWNSR-ABGSDEGCSA-N	C[C@H](CCC(=O)OC)[C@H]1CC[C@@H]2[C@@]1([C@H](C[C@H]3[C@H]2CC[C@H]4[C@@]3(CC[C@H](C4)O)C)O)C	TCMSP	Included
**110**	Bovis Calculus	ZINC01280365	ZESRJSPZRDMNHY-FJEWGBTGSA-N	C[C@]12CC[C@H]([C@]([C@H]1CC[C@@]3([C@@H]2CC=C4[C@]3(CC[C@@]5([C@@H]4CC(=C)CC5)C(=O)O)C)C)(C)CO)O	TCMSP	Included
**111**	Bovis Calculus	GCH	RFDAIACWWDREDC-FRVQLJSFSA-N	C1=CC(=C(C=C1/C=C\2/C(=O)C3=C(C=C(C=C3O2)O)O)O)O	TCMSP	Included
**112**	Pulsatillae Radix	Pinoresinol	HGXBRUKMWQGOIE-AFHBHXEDSA-N	C1=CC(=C(C=C1/C=C\2/C(=O)C3=C(O2)C=C(C=C3O[C@H]4[C@@H]([C@H]([C@@H]([C@H](O4)CO)O)O)O)O)O	TCMSP	Included
**113**	Pulsatillae Radix	androstane-3,11,17-triol	FNICIUSFFWRLFW-UUHZKGPGSA-N	C[C@]12CC[C@H]3[C@H]([C@H]1CC[C@@H]2C(=O)CO)CCC4=CC(=O)CC[C@]34C	TCMSP	Included

**114**	Pulsatillae Radix	cernuoside	ZZERRGHHDDWLEN-SKWVPNCFSA-N	COC1=C(C=CC(=C1)[C@@H]2[C@H]3CO[C@@H]([C@H]3CO2)C4=CC(=C(C=C4)O)OC)O	TCMSP	Included
**115**	Pulsatillae Radix	Aureusidin	WBEFUVAYFSOUEA-PQMHYQBVSA-N	C[C@H](CCC(=O)NCC(=O)O)[C@H]1CC[C@@H]2[C@@]1([C@H](C[C@H]3[C@H]2[C@@H](C[C@H]4[C@@]3(CC[C@H](C4)O)C)O)O)C	TCMSP	Included
**116**	Pulsatillae Radix	3beta,23-Dihydroxy-lup-20(29)-ene-28-O-alpha-L-rhamnopyranosyl-(1-4)-beta-D-glucopyranosyl(1-6)-beta-D-glucopyranoside_qt	JTOQVXAUPAUAAC-JMKRAKMLSA-N	N/A	TCMSP	Excluded
**117**	Pulsatillae Radix	ZINC01615307	HLBPOYVRLSXWJJ-NBHSMZAVSA-N	C1C[C@@H]2[C@@H]3C[C@@H](CN4[C@@H]3[C@@](CCC4)(CN2C(=O)C1)O)O	TCMSP	Included
**118**	Pulsatillae Radix	AIDS045703	HLBPOYVRLSXWJJ-UZCIPKQKSA-N	COC1=C(C=CC(=C1)C2=C(C(=O)C3=C(C=C(C=C3O2)O)O)O)O	TCMSP	Included
**119**	Pulsatillae Radix	5alpha,9alpha-Dihydroxymatrine	BYQQDLUCCAZYJO-ZZVJUGKHSA-N	COC1=CC(=CC(=C1OC)OC)[C@@H]2[C@@H]3[C@@H](CC4=C(C5=C(C=C24)OCO5)O)COC3=O	TCMSP	Included
**120**	Pulsatillae Radix, Sophorae Tonkinensis Radix	isorhamnetin	IZQSVPBOUDKVDZ-UHFFFAOYSA-N	COC1=CC(=CC(=C1OC)OC)[C@H]2[C@H]3[C@@H](CC4=C(C5=C(C=C24)OCO5)O)COC3=O	TCMSP	Included
**121**	Sophorae Tonkinensis Radix	daidzein	ZQSIJRDFPHDXIC-UHFFFAOYSA-N	C1=CC(=CC=C1C2=COC3=C(C2=O)C=CC(=C3)O)O	TCMSP	Included
**122**	Sophorae Tonkinensis Radix	formononetin	HKQYGTCOTHHOMP-UHFFFAOYSA-N	COC1=C(C=CC(=C1)C2=C(C(=O)C3=C(C=C(C=C3O2)O)O)O)O	TCMSP	Included

**123**	Sophorae Tonkinensis Radix	kaempferol	IYRMWMYZSQPJKC-UHFFFAOYSA-N	COC1=CC=C(C=C1)C2=COC3=C(C2=O)C=CC(=C3)O	TCMSP	Included
**124**	Sophorae Tonkinensis Radix	emodin	RHMXXJGYXNZAPX-UHFFFAOYSA-N	C1=CC(=CC=C1C2=COC3=CC(=CC(=C3C2=O)O)O)O	TCMSP	Included
**125**	Sophorae Tonkinensis Radix	Physcion	FFWOKTFYGVYKIR-UHFFFAOYSA-N	C1C[C@H]2CN3[C@H](CC=CC3=O)[C@@H]4[C@H]2N(C1)CCC4	TCMSP	Included
**126**	Sophorae Tonkinensis Radix	genistein	TZBJGXHYKVUXJN-UHFFFAOYSA-N	C1[C@@H]2[C@H](C3=C(O1)C=C(C=C3)O)OC4=CC5=C(C=C24)OCO5	TCMSP	Included
**127**	Sophorae Tonkinensis Radix	Inermine	HUKSJTUUSUGIDC-ZBEGNZNMSA-N	CC1=CC2=C(C(=C1)O)C(=O)C3=C(C2=O)C=C(C=C3O)O	TCMSP	Included
**128**	Sophorae Tonkinensis Radix	sophocarpine	AAGFPTSOPGCENQ-JLNYLFASSA-N	CC1=CC2=C(C(=C1)O)C(=O)C3=C(C2=O)C=C(C=C3O)OC	TCMSP	Included
**129**	Sophorae Tonkinensis Radix	Genistein 8-C-glucoside	HIWJJOYYZFELEZ-FFYOZGDPSA-N	C1=CC(=CC=C1C2=COC3=C(C2=O)C(=CC(=C3[C@H]4[C@@H]([C@H]([C@@H]([C@H](O4)CO)O)O)O)O)O)O	TCMSP	Included
**130**	Sophorae Tonkinensis Radix	Matsukaze lactone	NFNSSVSQSNJVCR-UHFFFAOYSA-N	C1=CC(=CC=C1C2=COC3=CC(=CC(=C3C2=O)O)O)O[C@H]4[C@@H]([C@H]([C@@H]([C@H](O4)CO)O)O)O	TCMSP	Included
**131**	Sophorae Tonkinensis Radix	Oxynarcotine	GLKPKUBRBLNOSC-UHFFFAOYSA-N	CNCCC1=CC2=C(C(=C1CC(=O)C3=C(C(=C(C=C3)OC)OC)C(=O)O)OC)OCO2	TCMSP	Included
**132**	Sophorae Tonkinensis Radix	8-O-methylretusin	GCQDBBHBZHZXQI-CYBMUJFWSA-N	COC1=C(C2=C(C=C1)C=CC(=O)O2)C3=C(C=C4C(=C3)C=CC(=O)O4)OC	TCMSP	Included
**133**	Sophorae Tonkinensis Radix	Sophoricoside	ISQRJFLLIDGZEP-CMWLGVBASA-N	COC1=CC=C(C=C1)C2=COC3=C(C2=O)C=CC(=C3OC)O	TCMSP	Included

**134**	Sophorae Tonkinensis Radix	Soyasaponin A6_qt	CDDWAYFUFNQLRZ-XUINOCJUSA-N	N/A	TCMSP	Included
**135**	Sophorae Tonkinensis Radix	Withaferine	DBRXOUCRJQVYJQ-PMMDLNQFSA-N	CC1=C(C(=O)O[C@H](C1)[C@@H](C)[C@H]2CC[C@@H]3[C@@]2(CC[C@H]4[C@@H]3C[C@@H]5[C@]6([C@@]4(C(=O)C=C[C@H]6O)C)O5)C)CO	TCMSP	Included
**136**	Sophorae Tonkinensis Radix	Abrisapogenol C	CSHFZVTYURMCRK-RPCMWVMTSA-N	C[C@@]12CC[C@H](C([C@H]1CC[C@@]3([C@@H]2CC=C4[C@]3(CC[C@@]5([C@@H]4C[C@]([C@@H]([C@H]5O)O)(C)CO)C)C)C)(C)C)O	TCMSP	Included
**137**	Sophorae Tonkinensis Radix	Sophojaponicin	VGSYCWGXBYZLLE-WFFYVNQPSA-N	C1[C@@H]2[C@H](C3=C(O1)C=C(C=C3)O)OC4=CC5=C(C=C24)OCO5	TCMSP	Included
**138**	Sophorae Tonkinensis Radix	Inermin	HUKSJTUUSUGIDC-BDJLRTHQSA-N	C1[C@H]2[C@@H](C3=C(O1)C=C(C=C3)O[C@H]4C([C@H]([C@@H](C(O4)CO)O)O)O)OC5=CC6=C(C=C25)OCO6	TCMSP	Included
**139**	Sophorae Tonkinensis Radix	7-hydroxy-2-(4-hydroxyphenyl)-8-[(2S,3R,4R,5S,6R)-3,4,5-trihydroxy-6-methylol-tetrahydropyran-2-yl]chromone	IAEWGSIPWPKEFT-UVPIGPOJSA-N	C1=CC(=CC=C1C2=CC(=O)C3=C(O2)C(=C(C=C3)O)[C@H]4[C@@H]([C@H]([C@@H]([C@H](O4)CO)O)O)O)O	TCMSP	Included
**140**	Sophorae Tonkinensis Radix	kudzusapogenol A	TVXOVTFPCNWYNK-HZJFQNLWSA-N	N/A	TCMSP	Included
**141**	Sophorae Tonkinensis Radix	Lupiwighteone	YGCCASGFIOIXIN-UHFFFAOYSA-N	C1C[C@@H]2[C@H]3CCCN4[C@H]3[C@@](CCC4)(CN2C(=O)C1)O	TCMSP	Included

**142**	Sophorae Tonkinensis Radix	sophoraflavone A_qt	IAEWGSIPWPKEFT-HXIIOJACSA-N	N/A	TCMSP	Included
**143**	Sophorae Tonkinensis Radix	7-hydroxy-2-[4-[(2S,3R,4S,5S,6R)-3,4,5-trihydroxy-6-methylol-tetrahydropyran-2-yl]oxyphenyl]chromone	GSZUGBAEBARHAW-YMQHIKHWSA-N	C1=CC(=CC=C1C2=CC(=O)C3=C(O2)C=C(C=C3)O)O	TCMSP	Included
**144**	Sophorae Tonkinensis Radix	7,4'-Dihydroxyflavone	LCAWNFIFMLXZPQ-UHFFFAOYSA-N	C1=CC(=CC=C1C2=CC(=O)C3=C(O2)C=C(C=C3)O)O[C@H]4[C@@H]([C@H]([C@@H]([C@H](O4)CO)O)O)O	TCMSP	Included
**145**	Sophorae Tonkinensis Radix	sophoranol	VQYBAEAOOJBSTR-NZBPQXDJSA-N	CC(=CCC1=C2C(=C(C=C1O)O)C(=O)C(=CO2)C3=CC=C(C=C3)O)C	TCMSP	Included
**146**	Sophorae Tonkinensis Radix	soyasapogenol A	CDDWAYFUFNQLRZ-VPXGNJILSA-N	C[C@]12CC[C@@H]([C@]([C@@H]1CC[C@@]3([C@@H]2CC=C4[C@]3(CC[C@@]5([C@H]4CC([C@H]([C@H]5O)O)(C)C)C)C)C)(C)CO)O	TCMSP	Included
**147**	Sophorae Tonkinensis Radix	subprogenin A	MCXBUCDXRNABAN-DQQHCUHRSA-N	N/A	TCMSP	Included
**148**	Sophorae Tonkinensis Radix	su+B1:B155bprogenin B	ZJZSSUXQPBSZBH-WFXZWOKGSA-N	N/A	TCMSP	Excluded
**149**	Moschus	Muscone	ALHUZKCOMYUFRB-UHFFFAOYSA-N	CC1CCCCCCCCCCCCC(=O)C1	Literature	Included
**150**	Fel Ursi	Ursodeoxycholic acid	RUDATBOHQWOJDD-UZVSRGJWSA-N	C[C@H](CCC(=O)O)[C@H]1CC[C@@H]2[C@@]1(CC[C@H]3[C@H]2[C@H](C[C@H]4[C@@]3(CC[C@H](C4)O)C)O)C	Literature	Included

**151**	Fel Ursi	TUDCA	BHTRKEVKTKCXOH-LBSADWJPSA-N	C[C@H](CCC(=O)NCCS(=O)(=O)O)[C@H]1CC[C@@H]2[C@@]1(CC[C@H]3[C@H]2[C@H](C[C@H]4[C@@]3(CC[C@H](C4)O)C)O)C	Literature	Included
**152**	Bovis Calculus	Cholic acid	BHQCQFFYRZLCQQ-OELDTZBJSA-N	C[C@H](CCC(=O)O)[C@H]1CC[C@@H]2[C@@]1([C@H](C[C@H]3[C@H]2[C@@H](C[C@H]4[C@@]3(CC[C@H](C4)O)C)O)O)C	Literature	Included
**153**	Bovis Calculus	Bilirubin	BPYKTIZUTYGOLE-IFADSCNNSA-N	CC1=C(NC(=C1CCC(=O)O)CC2=C(C(=C(N2)/C=C\\3/C(=C(C(=O)N3)C)C=C)C)CCC(=O)O)/C=C\\4/C(=C(C(=O)N4)C=C)C	Literature	Included
**154**	Bovis Calculus	Taurocholic acid	WBWWGRHZICKQGZ-HZAMXZRMSA-N	C[C@H](CCC(=O)NCCS(=O)(=O)O)[C@H]1CC[C@@H]2[C@@]1([C@H](C[C@H]3[C@H]2[C@@H](C[C@H]4[C@@]3(CC[C@H](C4)O)C)O)O)C	Literature	Included
**155**	Bovis Calculus	Glycocholic acid	RFDAIACWWDREDC-FRVQLJSFSA-N	C[C@H](CCC(=O)NCC(=O)O)[C@H]1CC[C@@H]2[C@@]1([C@H](C[C@H]3[C@H]2[C@@H](C[C@H]4[C@@]3(CC[C@H](C4)O)C)O)O)C	Literature	Included
**156**	Pulsatillae Radix Aucklandiae Radix Sophorae Tonkinensis Radix	Oleanolic acid	MIJYXULNPSFWEK-GTOFXWBISA-N	C[C@]12CC[C@@H](C([C@@H]1CC[C@@]3([C@@H]2CC=C4[C@]3(CC[C@@]5([C@H]4CC(CC5)(C)C)C(=O)O)C)C)(C)C)O	Literature	Included
**157**	Pulsatillae Radix	Ursolic acid	WCGUUGGRBIKTOS-GPOJBZKASA-N	C[C@@H]1CC[C@@]2(CC[C@@]3(C(=CC[C@H]4[C@]3(CC[C@@H]5[C@@]4(CC[C@@H](C5(C)C)O)C)C)[C@@H]2[C@H]1C)C)C(=O)O	Literature	Included
**158**	Pulsatillae Radix	Ergosterol	DNVPQKQSNYMLRS-APGDWVJJSA-N	C[C@H](/C=C/[C@H](C)C(C)C)[C@H]1CC[C@@H]2[C@@]1(CC[C@H]3C2=CC=C4[C@@]3(CC[C@@H](C4)O)C)C	Literature	Included

Table S6. Literature-supported targets manually incorporated into the V-pharmacopuncture (VP) pharmacological network (n = 14)

Target Gene	Linked VP Compound(s)	PMID(s)	Evidence Type	Mechanistic Role
**Axis 1: Inflammatory Signaling (TNF, IL6, IL1B, TLR4, STAT3)**				
**TNF**	Berberine, Baicalin, Baicalein, Muscone	28038998; 25550915; 26202808; 34748869	In vivo (mouse liver); Review	NF-κB pathway inhibition reducing TNF-α production; suppression of hepatic/systemic inflammatory response
**IL6**	Berberine, Baicalin, Muscone	28038998; 25550915; 34748869	In vivo (mouse liver); Review	Reduction of IL-6 levels via NF-κB blockade; attenuation of systemic inflammatory cascade
**IL1B**	Baicalein	26202808	In vivo (mouse)	Significant reduction of IL-1β mRNA expression; MAPK/ERK pathway inhibition
**TLR4**	Baicalin, Baicalein	25550915; 26202808	In vivo (mouse)	Down-regulation of TLR4 expression; early-stage blockade of upstream inflammatory signaling
**STAT3**	Wogonin	32566686	In vitro, In vivo (mouse)	Direct inhibition of STAT3 phosphorylation; blockade of inflammatory gene expression in hepatocytes
**Axis 2: Apoptosis Regulation (CASP3, BCL2, AKT1)**				
**CASP3**	Baicalin, TUDCA, Muscone	19960010; 12724520; 34748869	In vitro (hepatocyte, cardiac); Review	Inhibition of caspase-3 activation; blockade of the apoptotic execution step
**BCL2**	Baicalin, Muscone	19960010; 34748869	In vitro (hepatocyte, cardiac); Review	Up-regulation of BCL2 expression; enhancement of cell survival against apoptosis; increased Bcl-2/Bax ratio
**AKT1**	Berberine	23085271	In vitro (hepatocyte)	Activation of PI3K/AKT signaling; enhancement of hepatocyte viability against metabolic stress
**Axis 3: Oxidative Stress Defense (NFE2L2, HMOX1)**				
**NFE2L2**	Baicalin, Moschus, Oleanolic acid	28951767; 40288659; 19283895	In vivo (mouse liver); In vitro (HT22 cells)	Activation of Nrf2(NFE2L2)/Keap1 pathway; up-regulation of antioxidant and detoxifying enzyme expression; suppression of oxidative stress and ferroptosis
**HMOX1**	Baicalin, Moschus, Oleanolic acid	28951767; 40288659; 19283895	In vivo (mouse liver); In vitro (HT22 cells)	Induction of HO-1 expression downstream of Nrf2 activation; cytoprotective antioxidant effect; protection against chemical-induced hepatotoxicity; GPX4/SLC7A11 regulation
**Axis 4: Bile Acid Homeostasis (NR1H4/FXR)**				
**NR1H4 (FXR)**	UDCA, Cholic acid, TUDCA	25920087; 14652205	Review; In vivo (rat)	FXR (NR1H4) signaling pathway; bile acid-based hepatoprotective mechanisms; regulation of hepatobiliary bile acid transport

**Axis 5: Mitochondrial Quality Control — Ephedra-specific (PINK1, PRKN)**				
**PINK1**	Baicalin, TUDCA	40653429; 27699602	In vivo + In vitro (liver injury model); In vitro	Promotion of PINK1/Parkin-mediated mitophagy; clearance of damaged mitochondria; hepatoprotection against oxidative hepatotoxicity
**PRKN**	Baicalin, TUDCA	40653429; 27699602	In vivo + In vitro (liver injury model); In vitro	Enhancement of Parkin (PRKN) protein levels; strengthening of mitochondrial quality control system; protection against ephedrine-induced mitophagy dysregulation
**Axis 6: Fibrosis / Stress Signaling (TGFB1)**				
**TGFB1**	Berberine, Oxymatrine	36255058; 28849213	In vivo (rat), In vitro (HSC)	Inhibition of TGF-β1/Smads and NF-κB signaling; prevention of hepatic stellate cell activation and fibrotic remodeling

Each of the 14 targets was selected based on the following criteria: (a) experimental evidence of interaction with a specific VP compound in a hepatocyte-relevant or mechanistically analogous context; (b) a PubMed-indexed primary source or peer-reviewed review; and (c) documented involvement in at least one core pathological mechanism of drug-induced liver injury (inflammatory signaling, oxidative stress defense, apoptosis regulation, bile acid homeostasis, or mitochondrial quality control). PINK1 and PRKN were specifically incorporated based on primary experimental evidence linking ephedrine exposure to dysregulated PINK1–Parkin-mediated mitophagy — a mechanism absent from general DILI database predictions. NFE2L2 and HMOX1 evidence for Moschus reflects Nrf2/Keap1 pathway activation demonstrated in vitro; this mechanism is well-established as hepatoprotective in drug-induced oxidative liver injury contexts. These targets were incorporated to bridge the gap between database-derived predictions and established pathological mechanisms relevant to Ephedra-associated hepatotoxicity.

Abbreviations: VP, V-pharmacopuncture; UDCA, ursodeoxycholic acid; TUDCA, tauroursodeoxycholic acid; FXR, farnesoid X receptor; HO-1, heme oxygenase-1; NFE2L2/Nrf2, nuclear factor erythroid 2-related factor 2; HSC, hepatic stellate cell; NF-κB, nuclear factor kappa B; PMID, PubMed identifier.
